# Leveraging single-cell foundation models for accurate survival outcome prediction

**DOI:** 10.1093/bioadv/vbag076

**Published:** 2026-03-16

**Authors:** Wei Liu, Qiang Wang, Lin Long, Wei Wang

**Affiliations:** College of Science, Heilongjiang Institute of Technology, Harbin, Heilongjiang 150050, China; College of Surveying and Mapping Engineering, Heilongjiang Institute of Technology, Harbin, Heilongjiang 150050, China; Institute of Basic Medical Science, Cancer Research Center, Shantou University Medical College, Shantou, Guangdong 515041, China; College of Science, Heilongjiang Institute of Technology, Harbin, Heilongjiang 150050, China

## Abstract

**Motivation:**

Foundation models trained on large-scale single-cell transcriptomes can capture rich molecular representations of cellular states, yet their potential for cancer survival prediction from bulk RNA-seq data remains largely unexplored.

**Results:**

We applied the single-cell foundation model scFoundation to derive patient-level embeddings across 25 cancer types from TCGA and systematically evaluated their prognostic value under both cancer-specific and pan-cancer settings. To leverage complementary information, we developed an Embedding–Gene–Survival Prediction (EGSP) model that integrates foundation model embeddings with gene expression and clinical variables. EGSP achieved a mean concordance index (C-index) of 0.724 across cancers and exceeded 0.8 in seven cancer types, consistently outperforming single-modality models and existing multi-omics survival approaches. Comparative analyses showed that embeddings derived from pretrained scFoundation weights exhibited lower redundancy with gene expression while retaining complementary prognostic signals relative to pan-cancer fine-tuned embeddings. Explainable AI analyses further revealed that prognostic embeddings capture interpretable biological programs related to tumor differentiation, immune activity, and tumor-intrinsic growth, enabling transparent survival prediction at both cohort and patient levels. Overall, single-cell foundation model embeddings provide biologically meaningful and partially non-redundant survival signals that substantially improve bulk RNA-seq–based prognostic modeling.

**Availability and implementation:**

https://github.com/weiliu123/EGSP.

## 1 Introduction

Large-scale foundation models are transforming biomedicine, reshaping how we extract knowledge from diverse omics and clinical data, including single-cell transcriptomes (Wang *et al.* 2019, Ji *et al.* 2021, Chen *et al.* 2022, Elnaggar *et al.* 2022, Hao *et al.* 2024). By leveraging principles from natural language processing, these models learn universal representations from millions of single-cell transcriptomes, capturing higher-order dependencies and context at unprecedented resolution. Pioneering efforts such as scFoundation (Hao *et al.* 2024), scGPT (Cui *et al.* 2024), and GeneFormer (Theodoris *et al.* 2023) have demonstrated strong generalization across diverse downstream tasks. Importantly, although trained on scRNA-seq, their embeddings can also be transferred to bulk transcriptomic data (Hao *et al.* 2024), highlighting the potential of foundation models to serve as powerful priors for a wide range of biomedical applications. Mechanistically, this cross-modal transfer is commonly realized through fine-tuning pretrained representations for downstream tasks in machine learning. In particular, partial fine-tuning with selective layer unfreezing has emerged as an effective transfer learning strategy, allowing task adaptation while preserving generalizable representations learned during large-scale pretraining (Vrbančič and Podgorelec 2020, Davila *et al.* 2024). In cancer research, pan-cancer training can be viewed as a form of transfer learning that exploits shared molecular patterns across tumor types to improve robustness and generalization, especially in settings with limited cohort sizes (Hu *et al.* 2025, Zhang *et al.* 2025).

Accurate cancer survival prediction is central to precision oncology, guiding treatment decisions, risk stratification, and clinical trial design. Despite significant progress enabled by deep learning and multi-omics integration (Katzman *et al.* 2018, Cheerla and Gevaert 2019, Huang *et al.* 2020, Chai *et al.* 2021, Herrmann *et al.* 2020, Fan *et al.* 2023, Hu *et al.* 2025), survival prediction remains a challenging problem due to tumor heterogeneity, high-dimensional molecular features, and censored outcomes. Most existing approaches rely on raw molecular measurements or handcrafted representations, which may fail to capture higher-order biological structure embedded in large-scale transcriptomic data. In the foundation model era, however, only limited efforts have been devoted to leveraging such models for prognostic prediction. Mellors *et al.* (2024) applied GeneFormer embeddings for overall survival prediction in The Cancer Genome Atlas (TCGA) but only evaluated correlations with observed outcomes, while Liang *et al.* (2024) developed GeneBag for prognosis prediction but reframed survival as a 5-year binary classification, discarding valuable time-to-event information. Theus *et al.* (2024) trained Ridge-regularized Cox proportional hazards models (Ridge–Cox) (Simon *et al.* 2011) using embeddings from CancerFoundation and scGPT, yet these embeddings did not consistently outperform models trained directly on gene expression (Cui *et al.* 2024, Theus *et al.* 2024). Together, these studies suggest that while foundation model embeddings are promising, their prognostic utility and optimal integration strategies remain insufficiently understood. Inspired by evidence that incorporating gene–gene interaction information can enhance conventional survival modeling (Zhang *et al.* 2013, Ching *et al.* 2018, Wang and Liu 2020), we hypothesized that embeddings derived from foundation models—capable of capturing higher-order gene interactions—may encode novel prognostic signals beyond raw gene expression and thereby improve survival prediction. Nevertheless, whether embeddings inferred from bulk transcriptomic data are indeed associated with survival outcomes, and how to integrate them effectively with other modalities, remain open questions.

From a methodological standpoint, integrating heterogeneous molecular and clinical data can be framed as a feature fusion problem (Leng *et al.* 2022, Stahlschmidt *et al.* 2022, Duan *et al.* 2024, Guarrasi *et al.* 2025). Prior work in multi-omics survival modeling has explored early fusion through direct feature concatenation, as well as more complex late- or hybrid-fusion architectures (Duan *et al.* 2024, Zhao *et al.* 2024). Despite its conceptual simplicity, early feature fusion remains attractive due to its flexibility, interpretability, and effectiveness when fused representations encode complementary, non-redundant information (Stahlschmidt *et al.* 2022). We therefore posited that foundation model embeddings, when combined with raw gene expression and clinical variables, could serve as a complementary modality for survival prediction.

Here, we leverage scFoundation to derive patient-level embeddings from bulk transcriptomic data across 25 TCGA cancer types and systematically evaluate their prognostic value under cancer-specific and pan-cancer training strategies. By comparing different data modalities, transfer learning schemes, and integrative designs, we developed Embedding–Gene–Survival Prediction (EGSP), a foundation model–based framework that integrates embeddings, gene expression, and clinical features. EGSP achieved a mean C-index of 0.724 across 25 cancer types, with performance exceeding 0.8 in seven cancers. Our results demonstrate that embeddings derived from pretrained single-cell foundation models capture prognostic information that is partially non-redundant with raw gene expression, and that integrating these complementary signals through feature fusion substantially improves survival prediction. This work establishes a principled framework for applying foundation models to prognostic modeling and highlights their potential to bridge molecular representation learning and clinical outcome prediction in oncology.

## 2 Methods

### 2.1 Datasets

The RNA-seq counts data and clinical information (overall survival time, event, age, gender, and pTNM stage) were obtained from TCGA (http://cancergenome.nih.gov) via the GDC Data Portal. In line with the recommendations from Liu *et al.* (2018) and the methodology of Herrmann *et al.* (2020), we selected 25 cancer types with at least 50 samples and a minimum event ratio of 5% or 10 total events for survival prediction. This filtering strategy ensures that we could compute meaningful performance metrics. After removing duplicate samples within each cancer type and excluding those lacking survival information, we further excluded FFPE-preserved specimens because FFPE-associated RNA degradation and processing artifacts can systematically affect transcript abundance and increase technical noise. The final cohort comprised 8776 non-FFPE samples (3095 uncensored and 5681 censored) used for model development and evaluation (Fig. S1, available as supplementary data at *Bioinformatics Advances* online). Gene expression data for each sample were first normalized for library size using the scFoundation bulk-data pipeline (Hao *et al.* 2024), and then log(1 + *x*)—transformed at the gene level. Robustness of embedding extraction and downstream risk prediction to FFPE processing was assessed separately using matched FFPE and non-FFPE specimens available in TCGA.

### 2.2 Survival-related embeddings derivation

We first aligned the RNA-seq count data of each sample to the 19 264 genes modeled by scFoundation (Hao *et al.* 2024). To derive survival-related embeddings, samples within each cancer type were randomly divided into training (70%), evaluation (15%), and test (15%) sets. The gene-ranking step was conducted exclusively on the training samples: for each cancer type, we computed the univariate Cox proportional hazards *P*-value for each gene using the training set and selected the top *N_g_* genes with the smallest *P*-values as input to scFoundation, while setting the expression values of all other genes to zero. For bulk expression inputs, we followed the official scFoundation implementation (https://github.com/biomap-research/scFoundation), in which both the “target” (*T*) and “source” (*S*) total count tokens are set to the same value (*T *=* S*), corresponding to the total counts of the input genes. This configuration is conceptually appropriate for bulk data, where sequencing depth variation is not present.

### 2.3 Definition of model dimensions and design choices

In this study, *N_g_* denotes the number of survival-associated genes selected as input to scFoundation, *N_c_* the number of clinical variables incorporated into the survival model, and *N_e_* the dimensionality of the resulting scFoundation embeddings.

*N_g_* was treated as a tunable design parameter controlling the trade-off between prognostic relevance and computational efficiency. We evaluated *N_g_* ∈ {512, 1024, 2048}, which are comparable to the effective input lengths used during scFoundation pretraining (Hao *et al.* 2024) and correspond to <10% of the full gene set. As shown in the Section 3, these values yielded similar prognostic performance, with *N_g_* = 1024 providing a slightly more favorable balance between model performance and stability; this setting was therefore used in subsequent analyses.

*N_c_* corresponds to the number of available clinical variables and was not fixed across cancer types. Depending on data availability, clinical inputs included age and gender, with pTNM stage incorporated when available. Consequently, *N_c_* varied across cancer types, reflecting real-world heterogeneity in clinical annotation rather than a modeling constraint.

*N_e_* is determined by the pooling strategy used in scFoundation. When *pool_type* = “*all*,” embeddings have dimensionality *N_e_* = 3072, whereas *pool_type* = “*max*” yields *N_e_* = 768. The higher-dimensional embeddings were used when embeddings served as the primary representation, while the lower-dimensional configuration was adopted in integrative models to mitigate dominance of embedding features over gene expression inputs.

### 2.4 Architecture of scFoundation-based survival prediction models

**Figure 1:**
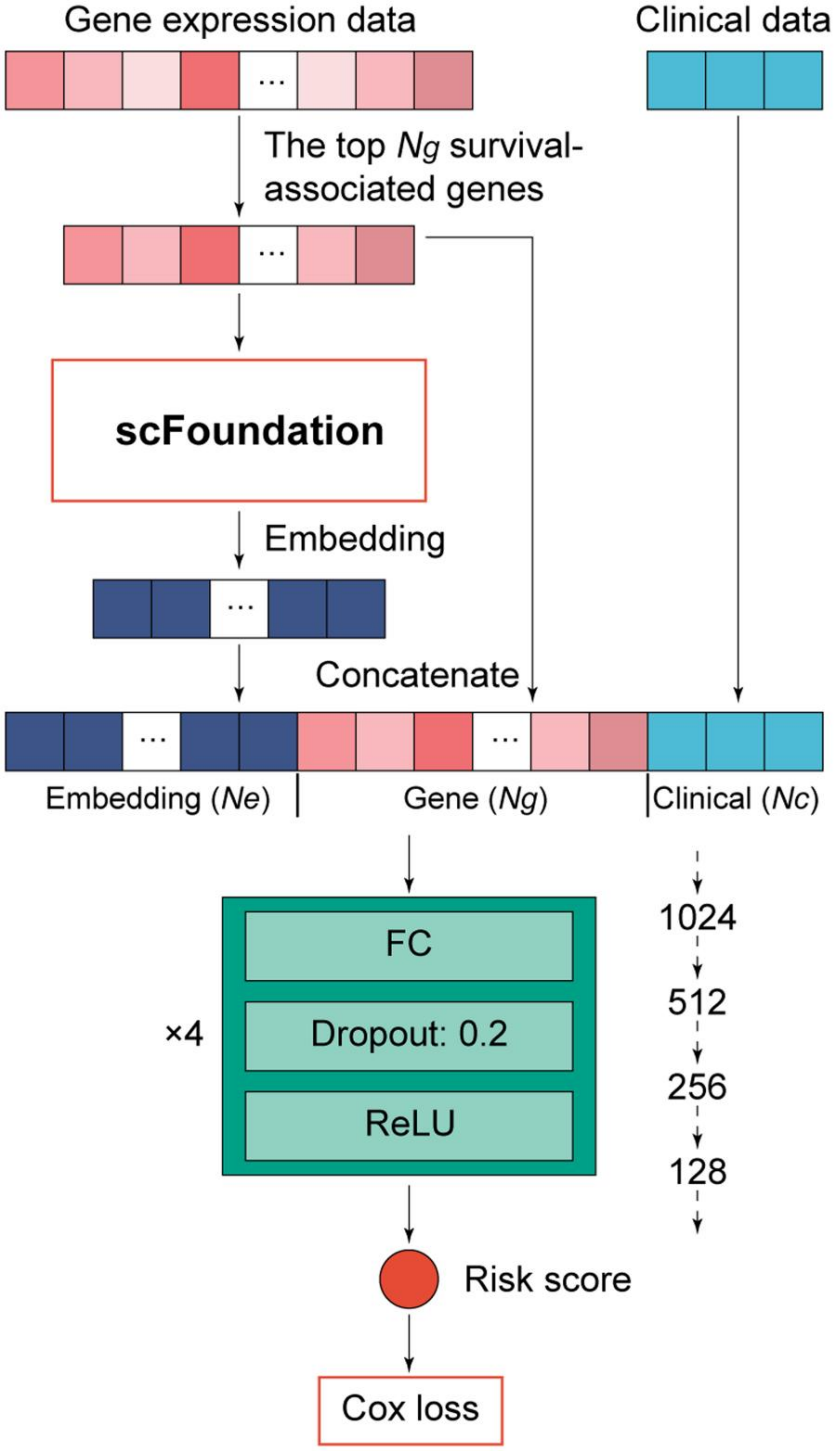
Architecture of scFoundation-based survival prediction models. *Ne*, *Ng*, and *Nc* denote the dimensionality of embedding, gene expression, and clinical features, respectively.

The overall architecture of the scFoundation-based survival prediction models is shown in Fig. 1. The inputs included gene expression data and clinical variables. The top *N_g_* survival-associated genes were fed into scFoundation to derive *N_e_*-dimensional embeddings. These embeddings were concatenated with mean-centered gene expression vectors and *N_c_*-dimensional clinical features, yielding an input vector of size *N_e_ + N_g_ + N_c_*. This representation-level integration implements a feature fusion strategy across molecular and clinical modalities. In its full multimodal configuration, this architecture corresponds to the EGSP model. The resulting input vector was passed to the survival prediction head, which consisted of multiple fully connected (FC) blocks. Each block comprised an FC layer, followed by dropout (rate = 0.2) and a ReLU activation. Based on empirical testing, we adopted four FC layers with dimensions 1024, 512, 256, and 128, which yielded consistently better performance compared with other configurations. The final output layer mapped the representation to a single risk score, which was optimized using the Cox partial likelihood loss function as defined by Katzman *et al.* (2018):


(1) $$l\left(\theta \right)=-\sum_{i=1}^{n}\delta_{i}\left[h_{\theta }\left(x_{i}\right)-\text{log}\;\left(\sum_{j=1}^{n}I\left(t_{j}\geq t_{i}\right)e^{h_{\theta }\left(x_{j}\right)}\right)\right]$$

where *t_i_*, *δ_i_*, and *x_i_* denote the survival time, event indicator, and input data of the *i*th patient, respectively. Specifically, *t_i_* corresponds to the observed survival time when *δ_i_* = 1 (event occurred), and to the right-censored time when *δ_i_* = 0. *h***_*θ*_**(·) represents the neural network model trained to predict risk scores, and *I*(·) is an indicator function taking value 1 if its argument is true and value 0 otherwise.

### 2.5 Model training and configurations

For each cancer type, patient samples were randomly divided into training, validation, and test sets at a ratio of 70%, 15%, and 15%, respectively. We adopted a stratified random partitioning strategy to maintain consistent censoring proportions across the training, validation, and test sets. This strategy was critical for enabling meaningful performance evaluation, as survival prediction metrics (e.g. C-index and iAUC) cannot be reliably computed when the number of observed events is too small. Models were trained on the training set to minimize the Cox loss function, with the validation set used for model selection. The learning rate was tuned over {2 × 10^−5^, 2 × 10^−4^, 2 × 10^−3^, 2 × 10^−2^}, the weight decay over {0.001, 0.01, 0.1, 0.25}, and the maximum gradient norm over {0.1, 1, 2, 5}. The optimizer was set to “adamw.” Training was performed for up to 200 epochs with an early-stopping strategy to prevent overfitting.

Depending on the data modalities and whether pan-cancer training was applied, we trained the following six models:

1. “Embed”: Used only the *Nₑ*-dimensional embeddings as input. In scFoundation, the parameter “*pool_type*” was set to “*all*,” resulting in embeddings of dimension *N_e_* = 3072.
2. “Embed + Clin”: Combined *N_e_* + *N_c_* features from embeddings and clinical variables (age, gender, pTNM), yielding a total of 3075 features.
3. “Embed-pan + Clin”: Used the same input as (II) but trained in a pan-cancer setting. Specifically, the training, validation, and test sets from 25 cancer types were merged to construct pan-cancer training, validation, and test cohorts. Models were trained on the pan-cancer training set to minimize the Cox loss function, with the pan-cancer validation set used for model selection. To capture pan-cancer signals, we unfroze *N_u_* (= 1, 2, 3, 4) transformer encoder layers in scFoundation, which modified the pretrained weights and consequently altered the derived embeddings, denoted as “Embed-pan.”
4. “mRNA + Clin”: Used *N_g_* + *N_c_* features from gene expression and clinical variables, totaling 1027 features.
5. “Embed-pan + mRNA + Clin” (EGSP-pan): An integrative survival prediction model combining Embed-pan, gene expression, and clinical variables. In our experiments, the 3072-dimensional Embed-pan often overshadowed the prognostic signal from gene expression (1024 dimensions) and yielded inferior performance. To alleviate this, we set *pool_type* = “*max*” in scFoundation, which reduced the embedding dimensionality to 768 and consistently improved prognostic prediction. The total input dimensionality was therefore 1795.
6. “Embed + mRNA + Clin” (EGSP): An integrative survival prediction model combining embeddings, gene expression, and clinical variables. It was identical to Model (V), except that the embeddings (Embed) were derived directly from the original pretrained scFoundation rather than from the pan-cancer-fine-tuned version (Embed-pan).

### 2.6 Computational environment and efficiency

All experiments were conducted on a single workstation equipped with an Intel i9-12900K CPU (128 GB RAM) and an NVIDIA RTX 3090 GPU (24 GB VRAM), running Windows 10, Python 3.12, PyTorch 2.5.1, and CUDA 12.4. We evaluated computational efficiency under two configurations. EGSP takes precomputed gene embeddings together with normalized gene expression features and clinical variables as input, enabling lightweight training and inference without requiring scFoundation during model optimization. In contrast, EGSP_End2End takes raw gene expression counts as input and extracts embeddings internally using a pretrained scFoundation encoder. Wall-clock time and peak GPU memory usage were measured using the Python *time* module and *torch.cuda.max_memory_allocated*(). EGSP showed minimal computational overhead, whereas EGSP_End2End incurred higher cost due to on-the-fly embedding extraction, but remained feasible on a single consumer-grade GPU even in the pan-cancer setting. Detailed timing and memory statistics are provided in Table S1, available as supplementary data at *Bioinformatics Advances* online.

### 2.7 Model evaluation

Model performance was primarily assessed using Harrell’s concordance index (C-index) (Harrell *et al.* 1996), which quantifies the concordance between predicted risk scores and observed survival outcomes and is widely used as a standard metric in survival analysis. A higher C-index indicates better discriminative ability of the model to correctly order patients by risk. The Harrell’s C-index was calculated using the *concordance_index* function from the *lifelines* (version 0.30.0) Python package or the *concordance.index* function from the *survcomp* (version 1.36.1) R package. In both the Python and R implementations, ties in survival times were handled by assigning a half-concordant pair (0.5×) to pairs with tied survival times, in line with standard practice in survival analysis. Specifically, in the R package *survcomp*, we set the *outx* parameter to *FALSE* to include ties in the calculation, ensuring consistency with the tie-handling mechanism in the Python *lifelines* package.

Harrell’s C-index is widely used in survival analysis, but it may exhibit upward bias under heavy censoring, particularly when censoring rates vary substantially across cohorts. To address this limitation, we also evaluated model performance using Uno’s C-index (Uno *et al.* 2011). Uno’s C-index accounts for censoring through inverse probability of censoring weighting (IPCW) and provides a more robust estimate of discriminative performance in the presence of censored survival data. Uno’s C-index was computed using the *concordance_index_ipcw* function from the *scikit-survival* (version 0.24.1) Python package or the *UnoC* function from the *survAUC* (version 1.3-0) R package. To improve numerical stability and avoid excessive influence from the tail of the follow-up distribution, the evaluation time horizon was restricted to *τ*, defined as the 90th percentile of the follow-up time in the training cohort, following common practice. Confidence intervals (CIs) for Uno’s C-index were estimated using stratified bootstrap resampling (2000 iterations), with event and censored cases resampled separately to ensure stable estimation under high censoring. For cancer types with very small test sets or limited numbers of comparable pairs, CI estimation could be unstable or degenerate; in such cases, confidence intervals were not reported and are indicated as NA.

In addition to C-index–based metrics, we computed the integrated time-dependent area under the curve (iAUC) (Haibe-Kains *et al.* 2008), which summarizes time-dependent receiver operating characteristic curves over the follow-up period and provides complementary information on the temporal stability of predictive performance. iAUC was computed using the *timeROC* R package (version 0.4). All performance metrics were evaluated on the held-out test set of each cancer type (or the pan-cancer test set, where applicable).

### 2.8 Redundancy assessment

Redundancy was assessed using mutual information (MI), which measures the amount of information shared between two random variables and thus reflects their dependency. Given two variables *X* (embeddings) and *Y* (gene expression), MI is defined as:


(2) $$I\left(X;Y\right)=\sum_{x\in X}\sum_{y\in Y}p\left(x,y\right)\;\text{log}\;\frac{p\left(x,y\right)}{p\left(x\right)p\left(y\right)},$$

where *p*(*x*, *y*) is the joint probability distribution of *X* and *Y*, and *p*(*x*) and *p*(*y*) are their marginal distributions. We calculated MI between embeddings and gene expression features to evaluate the extent to which embeddings retained or overlapped with raw molecular signals. In addition, we computed MI among embedding dimensions themselves to examine redundancy within the embedding space. All MI values were estimated using the R package *infotheo* (version 1.2.0.1), which discretizes continuous features and applies information-theoretic measures. For each cancer type, MI values were averaged across samples, providing a measure of redundancy either between embeddings and genes or among embedding dimensions. Lower MI indicates reduced redundancy and greater complementarity of information.

### 2.9 SHAP analysis

To interpret the feature importance and model predictions of the EGSP framework, we applied SHapley Additive exPlanations (SHAP) using the Python package *shap* (v0.50.0) (Lundberg and Lee 2017). For each cancer type, 50 samples were randomly selected from the training set to serve as the background distribution for SHAP value computation. Given that the EGSP model is based on a deep neural network architecture, we employed the *GradientExplainer* implementation to efficiently approximate SHAP values. Feature attributions were computed separately for the training, evaluation, and test datasets to assess consistency and generalizability across data splits.

For each sample, SHAP values quantify the contribution of individual input features (embeddings, gene expression features, and clinical variables) to the model-predicted risk score. Global feature importance was summarized as the mean absolute SHAP value across all samples within each cohort, while patient-level interpretability was visualized using summary plots and waterfall plots.

### 2.10 Pathway enrichment analysis

To investigate the biological pathways associated with embeddings, we performed Gene Set Enrichment Analysis (GSEA) (Subramanian *et al.* 2005) based on the correlation between each embedding feature and genome-wide gene expression profiles. For each embedding dimension of interest, Spearman correlation coefficients were calculated between the embedding values and gene expression levels across samples within each cancer cohort. Genes were then ranked in descending order according to their correlation coefficients, such that genes most positively correlated with the embedding were placed at the top of the ranked list, while negatively correlated genes were placed at the bottom.

GSEA was subsequently conducted using the *clusterProfiler* package (v4.16.0) (Xu *et al.* 2024) in R to identify pathways positively or negatively associated with each embedding. The analysis was performed with default parameters, using the Gene Ontology (GO) Biological Process databases as references. Enrichment results were evaluated based on the normalized enrichment score, and pathways with a false discovery rate <0.05 were considered statistically significant. Visualization of the top activated and suppressed pathways was performed using the *dotplot*() and *gseaplot2*() functions implemented in *clusterProfiler*.

### 2.11 Statistical analysis and visualization

Dimensionality reduction for visualization was performed using Uniform Manifold Approximation and Projection (UMAP) (McInnes *et al.* 2018) with default parameters, as implemented in the *umap-learn* (version 0.5.7) package. To assess concordance between paired measurements, Spearman’s rank correlation coefficient was used. Paired differences were evaluated using the Wilcoxon signed-rank test. All statistical tests were two-sided unless otherwise specified. Statistical analyses and visualizations were conducted using Python (version 3.12.11) and R (version 4.5.1).

## 3 Results

### 3.1 Prognostic power of scFoundation embeddings in survival prediction

**Figure 2:**
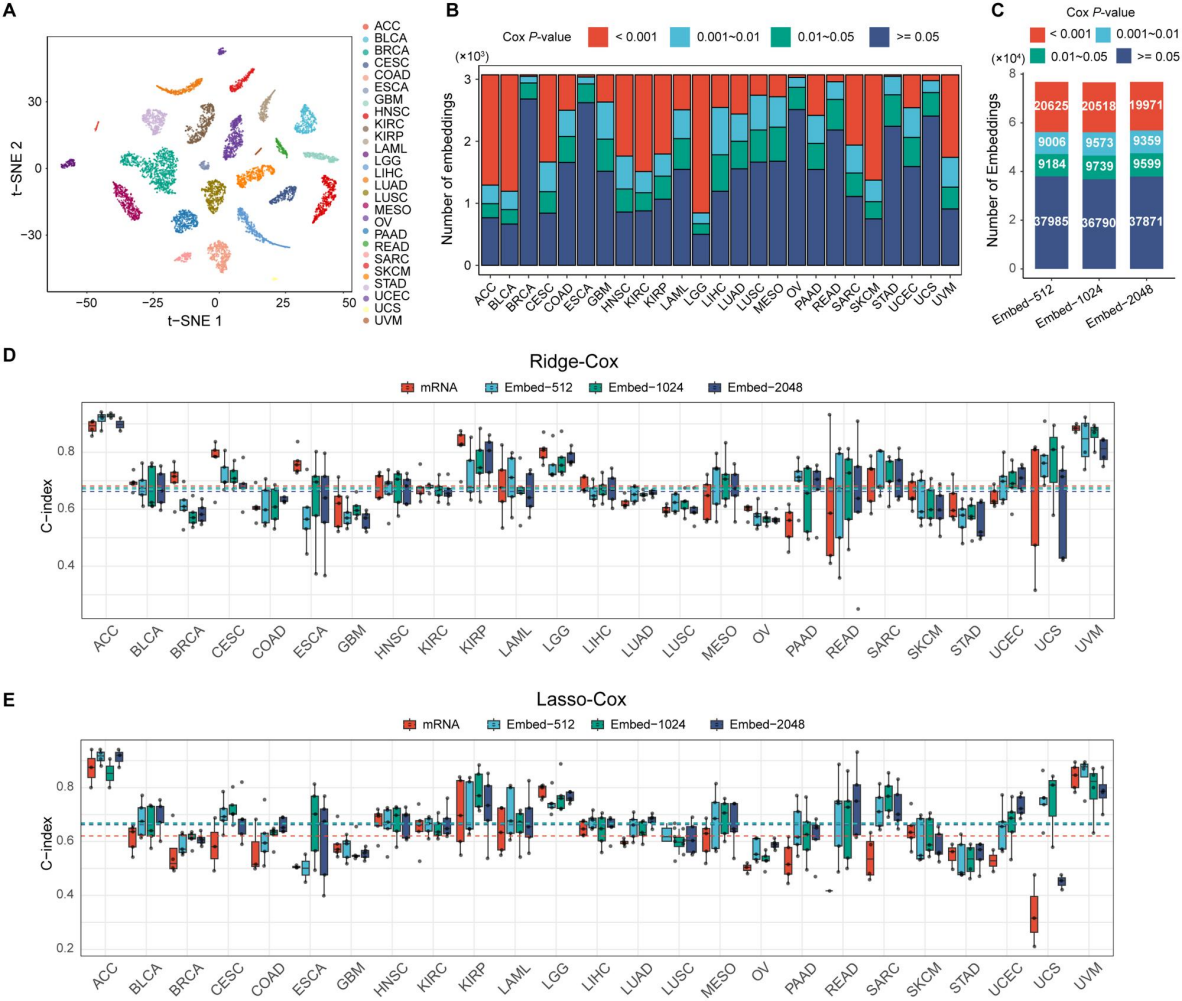
Evaluation of the prognostic power of scFoundation embeddings. (A) t-SNE visualization of scFoundation embeddings (Embed-1024) for 8776 patients, showing cancer type-specific clustering. (B) Distribution of Cox *P* values for Embed-1024 across cancer types. (C) Distribution of Cox *P* values for Embed-512, Embed-1024, and Embed-2048. (D and E) Effect of the number of input genes on the prognostic performance of embedding-based Cox models: (D) Ridge–Cox and (E) Lasso-Cox. Boxplots represent C-indices obtained from five-fold cross-validation. Dashed lines indicate the overall mean C-index across all cancer types for each feature set. mRNA, Cox models based on gene expression features; Embed-512, Embed-1024, and Embed-2048, embeddings derived from the top 512, 1024, and 2048 survival-associated genes, respectively.

To derive survival-related embeddings, we first ranked genes within each cancer type based on univariate Cox *P*-values computed from the training samples, and selected the top 1024 genes as input to scFoundation, generating patient-level embeddings (denoted Embed-1024). t-SNE visualization of the embeddings for all 8776 patients revealed cancer type–specific clustering (Fig. 2A), indicating that the embeddings capture intrinsic similarities within each cancer type as well as distinctions between different cancer types.

To evaluate their prognostic relevance, we performed univariate Cox regression analysis. On average, 51.9% of the embeddings were significantly associated with overall survival (Cox *P *< .05), ranging from 12.7% in BRCA to 83.7% in LGG, suggesting that Embed-1024 carries prognostic value (Fig. 2B). We also derived scFoundation embeddings using the top 512 and 2048 survival-associated genes (referred to as Embed-512 and Embed-2048, respectively). Across cancer types, the distributions of Cox *P*-values for Embed-512 and Embed-2048 closely resembled those of Embed-1024 (Fig. S2, available as supplementary data at *Bioinformatics Advances* online), and the total number of significant embeddings was comparable across all three settings, with Embed-1024 yielding slightly more (Fig. 2C).

We further evaluated the prognostic performance of scFoundation embeddings using Ridge–Cox and Lasso-Cox models (Simon *et al.* 2011). In the Ridge–Cox setting, 5-fold cross-validation showed that Embed-1024 achieved an average C-index of 0.680 ± 0.086 across 25 cancer types, comparable to Embed-512 (0.678 ± 0.083; Wilcoxon signed-rank test, *P *= .819) and slightly higher than Embed-2048 (0.667 ± 0.081; *P *= .024) (Fig. 2D and Table S2, available as supplementary data at *Bioinformatics Advances* online). The average C-indices of Embed-512 and Embed-1024 were also comparable to that of the gene expression–based Cox model (0.686 ± 0.089; *P *= .159 and .211, respectively). Since the Ridge–Cox model incorporates all gene features without feature selection, these results indicate that scFoundation embeddings effectively retain the prognostic information present in gene expression data.

For the Lasso-Cox model, the average C-indices for Embed-512, Embed-1024, and Embed-2048 were 0.667 ± 0.091, 0.670 ± 0.081, and 0.664 ± 0.091, respectively, all significantly higher than the gene expression–based Cox model (0.603 ± 0.120; *P *= 3.54 × 10^−6^, 2.17 × 10^−5^, and 2.05 × 10^−5^, respectively) (Fig. 2E and Table S3, available as supplementary data at *Bioinformatics Advances* online). These findings suggest that scFoundation embeddings provide more robust and stronger prognostic power compared to individual genes. Considering both the proportion of survival-associated embeddings and model performance, we selected Embed-1024 for subsequent analyses.

### 3.2 Pan-cancer training enhances prognostic modeling

To fully exploit the ability of scFoundation to capture gene–gene relationships and generate informative embeddings, we developed a deep learning–based survival prediction model built upon scFoundation. A survival prediction head consisting of four fully connected layers with 1024, 512, 256, and 128 nodes, respectively, was appended and optimized using a Cox loss function (see Section 2; Fig. 1). For each cancer type, patients were randomly divided into training, validation, and test sets (75%/15%/15%), with censoring proportions balanced across sets to ensure valid performance estimation. Using the top 1024 survival-associated genes as input, we trained the “Embed” model on the training set under the guidance of the validation set. However, when embeddings alone were used as predictors, the “Embed” model showed limited prognostic performance, achieving an average C-index of 0.624 across the 25 cancer-specific test sets (Table 1) and an average iAUC of 0.623 (Table S4, available as supplementary data at *Bioinformatics Advances* online).

**Table 1:** Performance comparison of different combinations of data modalities and training strategies on 25 cancer types using C-index (95% CI).^a^

Cancer	N (patients)	Censoring rate (%)	Embed	Embed + Clin	Embed-pan + Clin	mRNA + Clin	Embed-pan + mRNA + Clin			Embed + mRNA + Clin		
							Lasso–Cox	Ridge–Cox	EGSP-pan	Lasso–Cox	Ridge–Cox	EGSP
ACC	79	64.6	0.58 (0.13–0.93)	0.63 (0.15–0.94)	**0.83 (0.26–0.99)**	0.75 (0.21–0.97)	0.83 (0.57–1.00)	0.71 (0.35–1.00)	0.75 (0.21–0.97)	0.83 (0.57–1.00)	0.71 (0.31**–**1.00)	**0.83 (0.26–0.99)**
BLCA	402	56.0	0.62 (0.49–0.74)	0.66 (0.54–0.77)	0.67 (0.55–0.78)	**0.71 (0.59–0.81)**	0.64 (0.52–0.76)	0.69 (0.58–0.79)	0.71 (0.57–0.83)	0.63 (0.52–0.74)	0.69 (0.57**–**0.80)	0.69 (0.53–0.82)
BRCA	1079	68.1	0.49 (0.34–0.62)	0.70 (0.57–0.81)	0.67 (0.53–0.78)	0.74 (0.61–0.84)	0.74 (0.59–0.85)	0.66 (0.51–0.79)	0.68 (0.52–0.81)	0.74 (0.59–0.86)	0.64 (0.48**–**0.78)	**0.75 (0.63–0.85)**
CESC	304	76.3	0.74 (0.57–0.88)	0.72 (0.55–0.86)	0.72 (0.53–0.86)	0.72 (0.55–0.85)	0.49 (0.31–0.67)	0.65 (0.49–0.79)	0.73 (0.57–0.85)	0.55 (0.34–0.73)	0.66 (0.50**–**0.81)	**0.74 (0.58–0.87)**
COAD	455	77.6	0.66 (0.53–0.80)	0.65 (0.49–0.83)	0.66 (0.49–0.82)	0.70 (0.53–0.87)	0.77 (0.65–0.87)	0.67 (0.53–0.81)	0.64 (0.33–0.88)	**0.78 (0.65–0.88)**	0.64 (0.49**–**0.79)	0.69 (0.56–0.82)
ESCA	184	58.2	0.54 (0.26–0.75)	0.62 (0.41–0.80)	0.65 (0.45–0.81)	0.58 (0.31–0.78)	0.64 (0.45–0.81)	0.50 (0.34–0.66)	0.60 (0.40–0.78)	0.71 (0.54–0.86)	0.54 (0.36–0.71)	**0.81 (0.17–0.99)**
GBM	282	19.5	0.56 (0.42–0.70)	**0.66 (0.53–0.77)**	0.63 (0.51–0.75)	0.65 (0.52–0.77)	0.57 (0.43–0.70)	0.47 (0.32–0.61)	0.62 (0.49–0.74)	0.62 (0.48–0.74)	0.47 (0.33–0.61)	0.63 (0.49–0.75)
HNSC	519	57.4	0.66 (0.55–0.75)	0.66 (0.55–0.76)	0.62 (0.50–0.73)	0.66 (0.54–0.76)	0.51 (0.40–0.62)	0.38 (0.27–0.49)	0.67 (0.57–0.77)	0.53 (0.42–0.64)	0.48 (0.36–0.61)	**0.67 (0.56–0.77)**
KIRC	533	67.2	0.71 (0.60–0.81)	0.71 (0.60–0.82)	0.72 (0.61–0.84)	0.78 (0.67–0.87)	0.78 (0.69–0.87)	0.76 (0.66–0.84)	0.76 (0.65–0.86)	0.78 (0.68–0.86)	0.76 (0.67–0.85)	**0.82 (0.74–0.90)**
KIRP	287	84.7	0.91 (0.73–1.00)	0.90 (0.79–1.00)	0.91 (0.80–1.00)	0.91 (0.81–1.00)	0.72 (0.57–0.86)	0.95 (0.80–1.00)	0.88 (0.73–0.98)	0.67 (0.50–0.83)	0.91 (0.68–0.99)	**0.93 (0.84–0.99)**
LAML	140	37.9	0.66 (0.47–0.84)	0.69 (0.54–0.85)	0.72 (0.55–0.87)	0.78 (0.64–0.92)	0.33 (0.19–0.48)	0.49 (0.29–0.68)	0.78 (0.66–0.90)	0.46 (0.26–0.66)	0.62 (0.45–0.79)	**0.81 (0.69–0.93)**
LGG	513	75.6	0.81 (0.70–0.90)	0.76 (0.59–0.89)	0.79 (0.61–0.91)	0.81 (0.68–0.90)	0.20 (0.13–0.28)	0.51 (0.34–0.66)	0.83 (0.72–0.92)	0.45 (0.28–0.59)	0.79 (0.63–0.90)	**0.86 (0.73–0.95)**
LIHC	370	64.9	0.51 (0.37–0.66)	0.54 (0.41–0.67)	0.55 (0.43–0.68)	0.62 (0.49–0.76)	0.51 (0.39–0.65)	0.60 (0.46–0.74)	0.57 (0.42–0.72)	0.59 (0.48–0.71)	0.61 (0.47–0.75)	**0.68 (0.53–0.82)**
LUAD	497	63.8	0.72 (0.60–0.83)	0.68 (0.56–0.80)	0.66 (0.51–0.79)	0.77 (0.65–0.87)	0.70 (0.58–0.79)	0.66 (0.53–0.79)	**0.78 (0.67–0.87)**	0.71 (0.60–0.80)	0.75 (0.63–0.86)	0.77 (0.64–0.87)
LUSC	489	56.6	0.60 (0.48–0.71)	0.63 (0.50–0.74)	**0.67 (0.56–0.76)**	0.64 (0.52–0.75)	0.57 (0.45–0.69)	0.51 (0.37–0.65)	0.65 (0.51–0.75)	0.56 (0.44–0.68)	0.51 (0.37–0.64)	0.64 (0.51–0.75)
MESO	85	14.1	0.66 (0.41–0.86)	0.74 (0.52–0.92)	0.71 (0.45–0.91)	0.77 (0.55–0.94)	0.39 (0.16–0.70)	0.80 (0.66–0.92)	0.73 (0.49–0.93)	0.31 (0.13–0.60)	**0.80 (0.65–0.94)**	0.76 (0.49–0.94)
OV	419	37.7	0.45 (0.34–0.55)	0.61 (0.49–0.72)	0.58 (0.46–0.69)	0.59 (0.46–0.72)	0.48 (0.36–0.61)	0.53 (0.41–0.64)	0.65 (0.53–0.75)	0.50 (0.38–0.62)	0.52 (0.40–0.65)	**0.69 (0.57–0.79)**
PAAD	178	47.8	0.49 (0.33–0.66)	0.46 (0.30–0.64)	0.61 (0.44–0.81)	0.53 (0.37–0.70)	0.55 (0.36–0.74)	0.56 (0.37–0.75)	**0.62 (0.46–0.78)**	0.57 (0.39–0.75)	0.51 (0.29–0.72)	0.55 (0.37–0.74)
READ	164	84.1	0.54 (0.12–0.91)	0.57 (0.13–0.92)	**0.71 (0.20–0.96)**	0.50 (0.11–0.89)	0.43 (0.13–0.76)	0.50 (0.11–0.84)	0.70 (0.19–0.96)	0.45 (0.17–0.78)	0.46 (0.05–0.84)	0.59 (0.14–0.93)
SARC	259	62.2	0.62 (0.45–0.76)	0.47 (0.27–0.65)	0.52 (0.32–0.71)	0.61 (0.44–0.77)	0.47 (0.29–0.65)	0.50 (0.34–0.65)	**0.70 (0.49–0.85)**	0.46 (0.29–0.63)	0.41 (0.26–0.56)	0.61 (0.44–0.77)
SKCM	459	52.1	0.66 (0.54–0.76)	0.73 (0.63–0.82)	**0.74 (0.63–0.84)**	0.69 (0.58–0.79)	0.50 (0.38–0.62)	0.34 (0.23–0.47)	0.66 (0.56–0.76)	0.52 (0.39–0.65)	0.37 (0.26–0.48)	0.71 (0.61–0.82)
STAD	401	60.6	0.46 (0.34–0.59)	**0.65 (0.53–0.77)**	0.60 (0.48–0.73)	0.63 (0.49–0.76)	0.62 (0.49–0.74)	0.53 (0.40–0.67)	0.62 (0.50–0.72)	0.66 (0.52–0.78)	0.54 (0.41–0.69)	0.61 (0.46–0.77)
UCEC	541	83.2	0.69 (0.55–0.84)	0.56 (0.40–0.73)	0.59 (0.46–0.74)	**0.71 (0.59–0.85)**	0.71 (0.56–0.86)	0.70 (0.58–0.81)	0.59 (0.42–0.78)	0.70 (0.55–0.84)	0.62 (0.47–0.76)	0.69 (0.56–0.83)
UCS	57	38.6	0.46 (0.12–0.85)	0.50 (0.13–0.87)	0.69 (0.23–0.94)	0.39 (0.08–0.81)	0.19 (0.00–0.42)	0.69 (0.31–1.00)	**0.81 (0.31–0.98)**	0.27 (0.00–0.45)	0.65 (0.28–1.00)	0.58 (0.17–0.90)
UVM	80	58.8	0.82 (0.23–0.99)	0.73 (0.19–0.97)	0.73 (0.19–0.97)	0.82 (0.23–0.99)	0.82 (0.33–1.00)	0.73 (0.30–1.00)	0.64 (0.15–0.95)	0.77 (0.25–1.00)	0.73 (0.30–1.00)	**1.00 (NA)** ^b^
Mean±SD	–	–	0.624 ± 0.120	0.649 ± 0.098	0.678 ± 0.087	0.681 ± 0.113	0.567 ± 0.176	0.603 ± 0.139	0.694 ± 0.081	0.593 ± 0.146	0.616 ± 0.134	**0.724 ± 0.111**

a For each cancer type, the best result is shown in bold. Embed: embeddings derived directly from pretrained scFoundation weights; Embed-pan: embeddings from scFoundation fine-tuned by unfreezing the last three encoder layers in pan-cancer training; Clin, clinical data; mRNA, mRNA expression data; EGSP-pan, model integrating Embed-pan, gene expression, and clinical features; EGSP, model integrating Embed, gene expression, and clinical features.

b NA indicates that the confidence interval could not be reliably estimated due to the small number of events and/or high censoring rate in the test cohort.

To improve predictive accuracy, we incorporated clinical variables (age, gender, and pTNM stage) as additional input features, resulting in the “Embed + Clin” model. As expected, this model outperformed the embedding-only approach, with an average C-index of 0.649 (Table 1) and an average iAUC of 0.670 (Table S4, available as supplementary data at *Bioinformatics Advances* online).

Considering that pan-cancer training has been reported to enhance prognostic modeling (Cheerla and Gevaert 2019, Fan *et al.* 2023, Hu *et al.* 2025), we further constructed pan-cancer models (“Embed-pan” and “Embed-pan + Clin”) by combining training, validation, and test sets across all 25 cancer types. Beyond embeddings derived from the pretrained scFoundation weights, we sequentially unfroze the last one to four transformer encoder layers (out of 12 in total) to enable the model to capture pan-cancer-specific signals. Notably, unfreezing the last three layers yielded the highest performance among all settings, with “Embed-pan + Clin” achieving a C-index of 0.726 (95% CI, 0.702–0.751; Table 2) and an iAUC of 0.765 (95% CI, 0.738–0.792; Table S6, available as supplementary data at *Bioinformatics Advances* online), outperforming models with fewer or more unfrozen layers. Importantly, these results were obtained by training and evaluating a single pan-cancer model on pooled datasets across all cancer types, and are therefore not directly comparable to the cancer-type–specific averages reported in Table 1.

**Table 2:** Performance comparison of pan-cancer training across different numbers of unfrozen layers and input modalities, evaluated using C-index (95% CI).^a^

Unfrozen layers	Embed-pan		Embed-pan + Clin	
	Validation set	Test set	Validation set	Test set
None	0.730 (0.705–0.755)	0.706 (0.681–0.730)	0.745 (0.720–0.769)	0.720 (0.695–0.743)
−1	0.730 (0.706–0.756)	0.701 (0.676–0.726)	0.750 (0.727–0.773)	0.718 (0.692–0.742)
−1, −2	0.729 (0.705–0.754)	0.710 (0.685–0.735)	0.742 (0.717–0.766)	0.713 (0.687–0.738)
−1, −2, −3	0.735 (0.709–0.760)	0.705 (0.680–0.729)	0.748 (0.724–0.774)	**0.726 (0.702–0.751)**
−1, −2, −3, −4	0.736 (0.711–0.761)	0.706 (0.680–0.731)	0.740 (0.715–0.764)	0.716 (0.691–0.741)

a Bolded values indicate the highest performance in the test set. Performance in Table 2 was evaluated using a single pan-cancer model trained on pooled training sets and tested on a pooled pan-cancer test set across all 25 cancer types and is therefore not directly comparable to the cancer-type–specific mean performance reported in Table 1. Embed-pan denotes the pan-cancer model using Embed-pan as input, whereas Embed-pan + Clin additionally incorporates clinical variables. “Unfrozen layers” refers to the transformer encoder layers in scFoundation that were progressively unfrozen from the top (e.g. −1 = last layer, −1, −2 = last two layers, etc.).

Applying this optimized pan-cancer model to individual test sets improved the average C-index and iAUC to 0.678 and 0.705, respectively (Table 1 and Table S4, available as supplementary data at *Bioinformatics Advances* online), confirming that pan-cancer training indeed enhances prognostic performance. Importantly, “Embed-pan + Clin” demonstrated stronger robustness across all 25 cancer types, rescuing performance in cancer types where embedding-only models performed no better than or only marginally above random guessing, such as PAAD (0.61 versus 0.49), READ (0.71 versus 0.54), and UCS (0.69 versus 0.46).

### 3.3 Integrating embeddings and gene expression (EGSP) improves prognostic performance

Although “Embed-pan + Clin” stabilized performance, it performed only comparably to the Ridge–Cox model built on Embed-1024 features (Table S2, available as supplementary data at *Bioinformatics Advances* online). Moreover, the “mRNA + Clin” model, built by replacing embeddings with gene expression, achieved an average C-index of 0.681 and iAUC of 0.698 (Table 1 and Table S4, available as supplementary data at *Bioinformatics Advances* online), marginally surpassing “Embed-pan + Clin” (C-index = 0.678) and underscoring the prognostic value of raw gene expression. This observation is consistent with the findings of Theus *et al.* (2024), who showed that embeddings from the single-cell foundation models CancerFoundation and scGPT (Cui *et al.* 2024) generally do not outperform baselines trained directly on gene expression across most cancer types. To harness both raw molecular signals and pan-cancer-learned representations, we developed EGSP-pan, which integrates Embed-pan, gene expression, and clinical features (Fig. 1). EGSP-pan further improved overall performance (C-index = 0.694; iAUC = 0.731) and achieved the highest accuracy in LUAD, PAAD, SARC, and UCS (Table 1 and Table S6, available as supplementary data at *Bioinformatics Advances* online).

However, in 15 of 25 cancer types, EGSP-pan underperformed relative to “Embed-pan + Clin” or “mRNA + Clin,” likely due to redundancy between Embed-pan—embeddings derived from scFoundation with the last three transformer encoder layers unfrozen and fine-tuned through pan-cancer training—and gene expression. As an alternative strategy, we developed the EGSP model by integrating gene expression and clinical features with Embed—embeddings derived directly from pretrained scFoundation weights. Strikingly, EGSP achieved the best overall performance, with a mean C-index of 0.724 and iAUC of 0.771 (Table 1 and Table S4, available as supplementary data at *Bioinformatics Advances* online), surpassing 0.8 in seven cancer types (ACC, ESCA, KIRC, KIRP, LAML, LGG, and UVM). Across all 25 cancer types, EGSP significantly outperformed “Embed-pan + Clin” (Wilcoxon signed rank test, *P *= 8.52 × 10^−3^), “mRNA + Clin” (*P *= 2.67 × 10^−3^), and EGSP-pan (*P *= 4.63 × 10^−2^).

Because Harrell’s C-index can be upwardly biased under heavy censoring and censoring rates vary substantially across TCGA cancer types, we additionally evaluated performance using Uno’s C-index and reported cohort size and censoring rate for each cancer type (Table 1 and Table S5, available as supplementary data at *Bioinformatics Advances* online). Across models, Uno’s C-index yielded consistent conclusions while generally providing more conservative estimates (e.g. EGSP: mean UnoC = 0.692 ± 0.133 versus mean Harrell C-index = 0.724 ± 0.111). For cancer types with small effective test cohorts or few observed events, extreme C-index values (e.g. C-index = 1.0 in UVM) should be interpreted cautiously, as they may arise from a limited number of comparable pairs; accordingly, confidence intervals were unstable or could not be reliably estimated and are marked as NA (Table S5, available as supplementary data at *Bioinformatics Advances* online).

For completeness, we further evaluated traditional regularized Cox models (lasso and ridge) using the full feature space comprising embeddings, raw gene expression, and clinical variables. Both models exhibited inferior performance compared with models using embeddings or gene expression alone (mean C-index: 0.567–0.616; Table 1 and Tables S4 and S5, available as supplementary data at *Bioinformatics Advances* online), likely due to redundancy and collinearity among high-dimensional molecular features. Notably, under the same linear modeling framework, combinations based on Embed consistently outperformed those using Embed-pan, suggesting that embeddings derived from frozen scFoundation weights retain more complementary prognostic information when integrated with gene expression.

Because FFPE samples were excluded during model training, we performed a sensitivity analysis using TCGA cases with matched FFPE and non-FFPE specimens to assess the robustness of scFoundation-derived embeddings and downstream risk prediction. Across three representative cancer types (BRCA, COAD, and LUAD), UMAP visualization revealed a systematic shift between FFPE and non-FFPE embeddings, while largely preserving pairwise correspondence between matched samples (Fig. S3A, available as supplementary data at *Bioinformatics Advances* online). Importantly, EGSP-predicted risk scores derived from FFPE samples were strongly correlated with those from matched non-FFPE samples (Spearman’s *ρ*  =  0.74–0.84; Fig. S3B, available as supplementary data at *Bioinformatics Advances* online), indicating preserved risk ranking. Moreover, paired differences in predicted risk scores were centered near zero with no evidence of systematic directional shift (Wilcoxon signed-rank test, *P *= 0.68–1.00; Fig. S3C, available as supplementary data at *Bioinformatics Advances* online). Together, these results suggest that FFPE-associated technical variation primarily increases noise without introducing consistent bias in EGSP-based survival risk prediction.

### 3.4 Non-redundant risk embeddings drive EGSP performance

**Figure 3:**
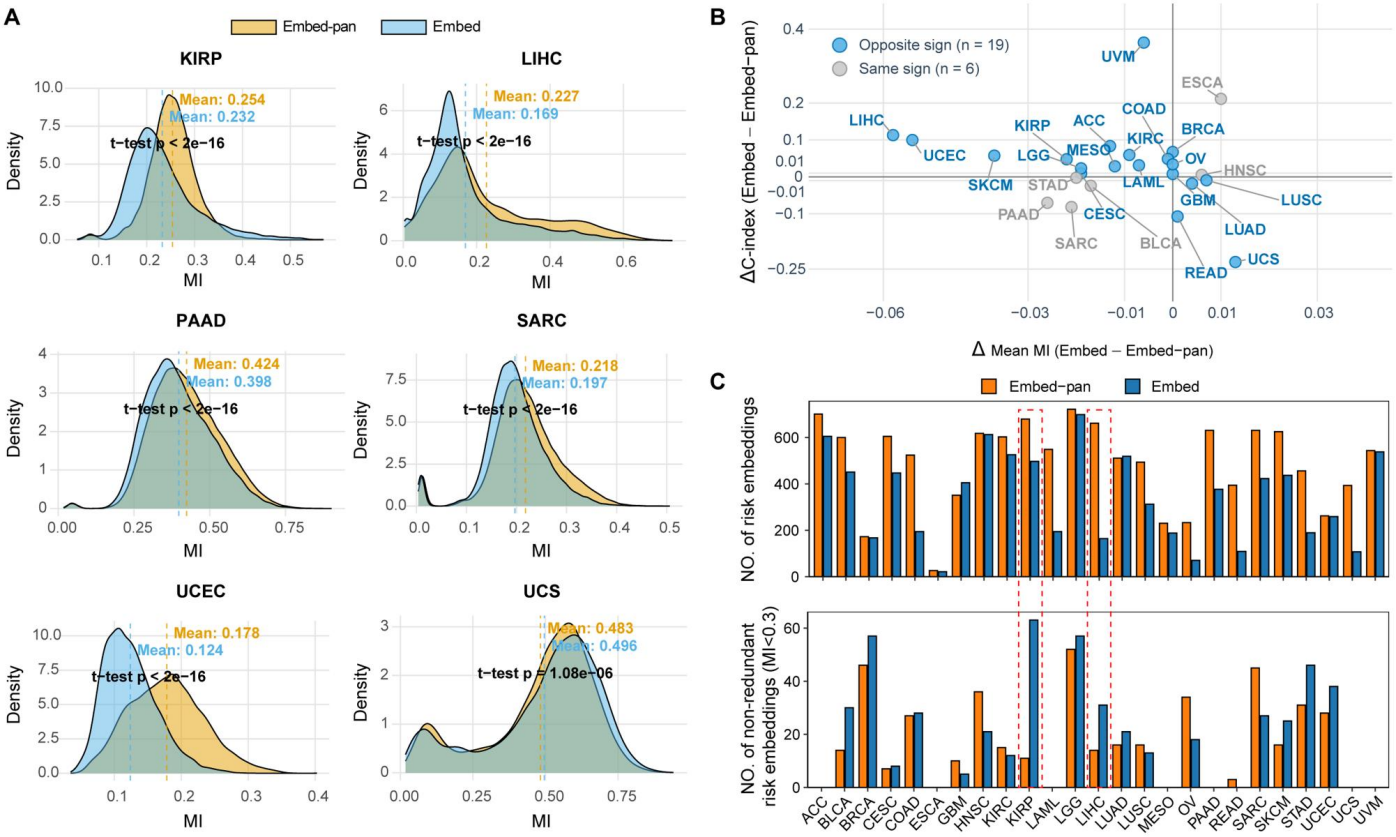
Evaluation of redundancy between embeddings and gene expression. (A) Distribution of mutual information (MI) between embeddings and gene expression for six cancer types. (B) Comparison of MI differences with C-index differences. Points corresponding to cases where lower MI coincides with higher EGSP performance are indicated. (C) Numbers of risk embeddings (top) and non-redundant risk embeddings (bottom). Embed: embeddings derived directly from pretrained scFoundation weights; Embed-pan: embeddings from scFoundation fine-tuned by unfreezing the last three encoder layers in pan-cancer training.

Here, “risk embeddings” refer to embedding features significantly associated with survival outcomes, identified by univariate Cox proportional hazards analysis (*P *< .05). This definition parallels the concept of “risk genes” commonly used in survival analysis. The number of risk embeddings varies across cancer types because the input gene sets and their expression profiles are cancer-specific, resulting in distinct sets of prognostic embeddings that reflect differences in the underlying molecular determinants of survival. To explore why EGSP outperformed EGSP-pan, we quantified redundancy between risk embeddings and risk genes (both defined by Cox *P *< .05) using MI. Across 16 cancer types, the embeddings from EGSP (Embed) generally exhibited lower MI values than those from EGSP-pan (Embed-pan) (Fig. 3A and B;  Fig. S4, available as supplementary data at *Bioinformatics Advances* online). Moreover, in 76% (19/25) of cancer types, a lower MI was accompanied by a higher EGSP C-index (Fig. 3B), indicating that EGSP benefited from reduced redundancy between embeddings and gene expression.

We further quantified non-redundant risk embeddings, defined as those with MI < 0.3 with all other embeddings and genes. Although Embed-pan contained more risk embeddings overall, it consistently provided fewer non-redundant ones than Embed (Fig. 3C). For instance, in KIRP—where EGSP reached the highest C-index (0.93)—Embed-pan showed higher average MI with genes (0.254 versus 0.232, *t*-test *P *< 2 × 10^−16^; Fig. 3A) and included more total risk embeddings, yet far fewer were non-redundant (Fig. 3C). A similar pattern was observed in LIHC. These results highlight non-redundant risk embeddings as key drivers of EGSP’s superior performance.

### 3.5 Comparison with previous work

We compared EGSP with seven representative multi-omics survival prediction models on TCGA (Cheerla and Gevaert 2019, Huang *et al.* 2020, Chai *et al.* 2021, Fan *et al.* 2023, Hu *et al.* 2025). As different studies relied on distinct sample sets due to missing modalities, direct head-to-head comparison was not feasible; we therefore present only a rough benchmark against reported C-index values. Using only gene expression and clinical features, EGSP improved the average C-index by 14.8% over Huang *et al.*’s (2020) gene expression–only model, and by 8.5% and 22.0% over the models of Fan *et al.* (2023) and Cheerla and Gevaert (2019), who also integrated gene expression and clinical features (Table S7, available as supplementary data at *Bioinformatics Advances* online). EGSP also outperformed multi-omics models: compared with Fan *et al.*’s (2023) pan-cancer model integrating gene expression, clinical data, and CNV, and the CATfusion (Hu *et al.* 2025) and DCAP (Chai *et al.* 2021) models incorporating six and four modalities, EGSP achieved average gains of 9.5%, 6.9%, and 6.1%, respectively. The only exception was Cheerla and Gevaert’s (2019) model, which integrated clinical, mRNA, miRNA, and whole-slide images (WSI) data and surpassed EGSP overall (Table S7, available as supplementary data at *Bioinformatics Advances* online). Notably, EGSP achieved the highest C-index in cancers such as KIRC, KIRP, and LAML, but showed room for improvement in PAAD, STAD, and UCEC. These observations suggest that incorporating additional modalities such as WSI may further enhance the prognostic performance of EGSP, warranting future investigation.

### 3.6 SHAP analysis reveals cross-cancer stability and interpretability of prognostically important embeddings

**Figure 4:**
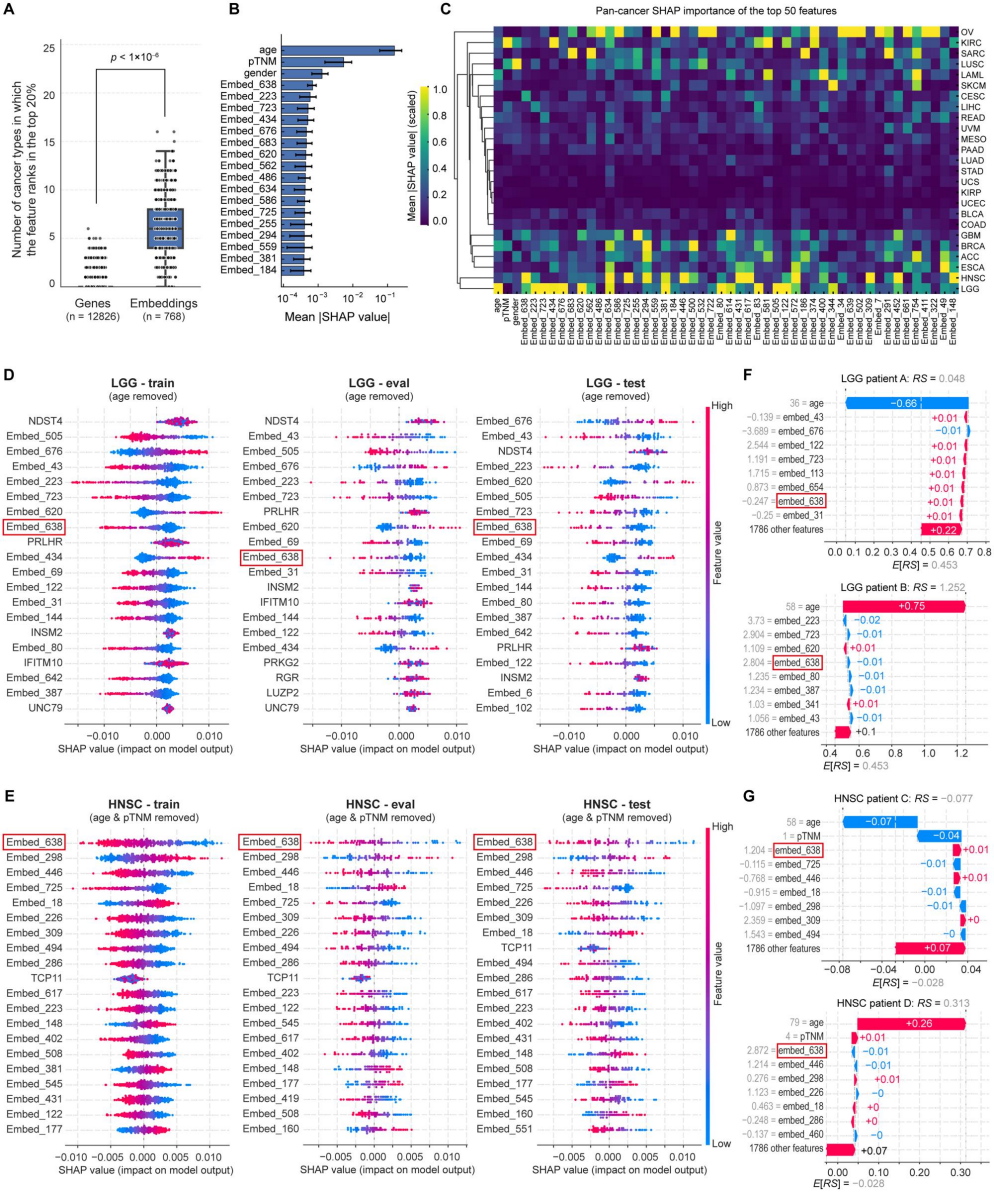
SHAP analysis of prognostically important embeddings in the EGSP model. (A) Cross-cancer stability of feature importance between genes and embeddings. Each dot represents a feature; the y-axis indicates the number of cancer types in which the feature ranks within the top 20% by mean absolute SHAP value. *P* value was calculated using a one-sided Wilcoxon rank-sum test. (B) Top 20 features ranked by mean absolute SHAP values across 25 cancer types. Bars represent average pan-cancer importance, with error bars indicating cancer-specific variability. (C) Heatmap of pan-cancer mean absolute SHAP values for the top 50 features, selected based on their overall ranking across 25 cancer types. (D) SHAP summary plots showing the top 20 most influential features in the LGG cohort for the training (left), evaluation (middle), and test (right) datasets. Each dot represents one sample, with a color gradient indicating the relative feature value (from lower to higher values). For clarity, the *age* feature was excluded; the complete feature set is provided in the Fig. S5A, available as supplementary data at *Bioinformatics Advances* online. (E) SHAP summary plots for the HNSC cohort, with *age* and *pTNM* features removed for visualization. The complete feature set is provided in the Fig. S5B, available as supplementary data at *Bioinformatics Advances* online. All other details are as described in panel (D). (F) Waterfall plots illustrating SHAP value contributions to the predicted risk score (*RS*) for two representative LGG patients. Each bar indicates the impact of a feature on an individual’s *RS* relative to the cohort mean prediction (*E*[*RS*]). (G) Waterfall plots for two representative HNSC patients, showing individual-level SHAP value decomposition of predicted *RS*, as in panel (F).

To address the interpretability and potential biological relevance of the proposed EGSP model, we applied explainable artificial intelligence (XAI) techniques based on SHAP (Lundberg and Lee 2017) to quantify feature contributions to survival prediction across cancer types. We first compared the cross-cancer stability of feature importance between gene and embedding features, with importance quantified by SHAP values from the EGSP model. For each feature, we calculated the number of cancer types in which it ranked within the top 20% by mean absolute SHAP value. Embeddings exhibit significantly higher pan-cancer stability than gene-level features, as evidenced by a greater number of cancer types in which they rank among the top contributors to survival prediction (Wilcoxon rank-sum test, *P *< 1 × 10^−6^; Fig. 4A). This indicates that embeddings capture prognostically relevant information that is more stable across cancer types.

Next, we ranked features based on their average absolute SHAP values across 25 cancer types to identify the most influential predictors at the pan-cancer level. Apart from clinical features, embedding features dominated the top ranks, including Embed-638, Embed-223, and others (Fig. 4B). The heatmap in Fig. 4C displays the mean absolute SHAP values of the top 50 embeddings across all 25 cancer types. To further investigate the robustness of feature importance within individual cancer cohorts, we focused on Embed-638, which ranked the highest, for detailed analysis. Embed-638 exhibited the highest mean absolute SHAP values in LGG and HNSC. In the LGG cohort, the top 20 influential features, including Embed-638, exhibited highly consistent SHAP distributions across the training, evaluation, and test datasets (Fig. 4D and Fig. S5A, available as supplementary data at *Bioinformatics Advances* online), indicating stable feature attribution and limited overfitting. A similar pattern was observed in the HNSC cohort (Fig. 4E and Fig. S5B, available as supplementary data at *Bioinformatics Advances* online), where Embed-638 consistently ranked first across all three datasets, further supporting the reproducibility of SHAP-based feature importance across independent data splits.

Finally, we visualized the individual-level SHAP value contributions to the predicted risk score (*RS*) for two representative patients from the LGG and HNSC cohorts (Fig. 4F and G). These waterfall plots illustrated how Embed-638 influenced the patients’ individual risk scores relative to the cohort mean prediction (*E*[*RS*]). In LGG, Embed-638 contributes +0.01 to the predicted *RS* of patient A (top), but −0.01 for patient B (bottom) (Fig. 4F). In HNSC, Embed-638 also showed differential contributions to patients C and D, highlighting the heterogeneous effects of this embedding dimension across individuals. These individual-level insights emphasize the potential of XAI methods to uncover meaningful biological associations between embedding dimensions and clinical outcomes.

To investigate the apparent performance gains observed in cancer types with limited sample sizes, such as ESCA and UVM, we performed a focused SHAP-based feature attribution analysis (Figs. S6–S9, available as supplementary data at *Bioinformatics Advances* online). First, we examined the top-ranked features across the training, evaluation, and test splits. In ESCC, the top 20 features in both the EGSP and EGSP-pan models were predominantly gene-related features, whereas in UVM, the top 20 features of the EGSP model were mainly gene features, while those of the EGSP-pan model were largely embedding-derived features (Figs S8 and S9, available as supplementary data at *Bioinformatics Advances* online). This pattern highlights distinct feature dependence across cancer types and suggests that the observed performance improvement is not driven by a small subset of dominant embedding features. We next quantified the relative contributions of embedding, gene, and clinical features at the feature-type level. Notably, EGSP consistently assigned a higher proportion of total SHAP contribution to clinical features compared with EGSP-pan across all data splits, whereas EGSP-pan shifted predictive weight toward embedding features (Fig. S10A, available as supplementary data at *Bioinformatics Advances* online). This redistribution was reproducible in both ESCA and UVM (Fig. S11A, available as supplementary data at *Bioinformatics Advances* online). Per-sample SHAP analyses further confirmed that these differences reflected systematic shifts in feature utilization rather than effects driven by a small number of outlier samples (Figs S10B–E and S11B–E, available as supplementary data at *Bioinformatics Advances* online). Together, these results suggest that the high apparent performance of EGSP in ESCA and UVM is primarily associated with stronger utilization of cohort-specific clinical information, while pan-cancer fine-tuning increases reliance on embedding features.

### 3.7 Prognostic relevance and functional interpretation of Embed-638

**Figure 5:**
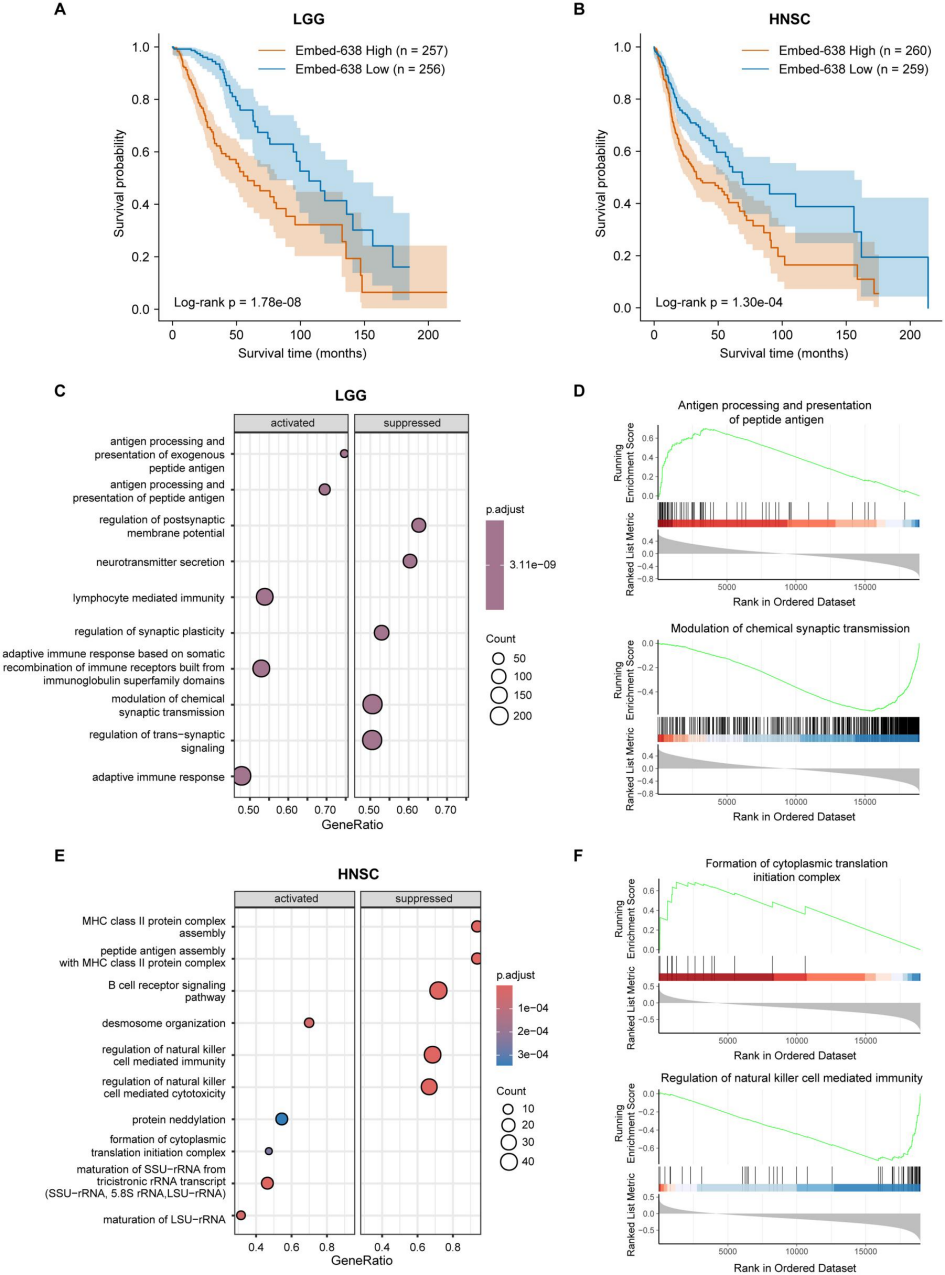
Prognostic relevance and functional characterization of Embed-638 in LGG and HNSC. (A and B) Kaplan–Meier curves for the LGG (A) and HNSC (B) cohorts, in which patients were stratified into high and low Embed-638 groups using the median value as the cutoff. (C) GSEA results for the LGG cohort based on Spearman correlations between Embed-638 and genome-wide gene expression. The top five activated (left) and suppressed (right) pathways are shown; bubble size represents gene count and color indicates adjusted *P*-value. (D) Representative enrichment plots for LGG showing an activated pathway (top) and a suppressed pathway (bottom). (E) GSEA results for the HNSC cohort, generated as in panel (C), showing the top five activated and suppressed pathways. (F) Representative enrichment plots for HNSC showing an activated pathway (top) and a suppressed pathway (bottom).

To investigate the prognostic significance of Embed-638, we stratified patients into high and low groups based on the median value and performed Kaplan–Meier survival analysis. In both LGG and HNSC, patients with higher Embed-638 values exhibited significantly worse overall survival (log-rank *P *= 1.78 × 10^−8^ for LGG and 1.30 × 10^−4^ for HNSC), highlighting its role as a prognostic marker across cancer types (Fig. 5A and B).

To gain mechanistic insight, we correlated Embed-638 with genome-wide gene expression profiles and performed GSEA analysis. In LGG, higher Embed-638 values were associated with strong negative enrichment of neuronal and synaptic pathways, including modulation of chemical synaptic transmission and related neuronal signaling processes (Fig. 5C and D and Table S8, available as supplementary data at *Bioinformatics Advances* online). These results indicate a marked depletion of neuron-like transcriptional programs in tumors with high Embed-638. In contrast, positive enrichment was observed for immune-related pathways, including adaptive immune response, antigen processing and presentation, and lymphocyte-mediated immunity, suggesting increased immune activity in high Embed-638 tumors (Fig. 5C and D and Table S8, available as supplementary data at *Bioinformatics Advances* online). These findings suggest that Embed-638 reflects a transcriptional shift from neuronal differentiation programs to an immune-enriched tumor microenvironment, consistent with previous reports that LGG retaining neuronal lineage features are associated with more favorable prognosis, whereas loss of neuronal differentiation and increased immune infiltration characterize more aggressive disease states (Guo *et al.* 2020, Song *et al.* 2020, Wu *et al.* 2020, Han *et al.* 2021, Luo *et al.* 2021).

In HNSC, GSEA revealed Embed-638’s strong association with the activation of tumor-intrinsic programs related to ribosome biogenesis, RNA processing, and protein homeostasis, such as ribosomal RNA maturation and formation of the cytoplasmic translation initiation complex (Fig. 5E and F and Table S9, available as supplementary data at *Bioinformatics Advances* online). These pathways are hallmark features of enhanced translational capacity, proteostasis remodeling, and proliferative potential, which have been linked to aggressive tumor behavior and adverse clinical outcomes in HNSC (Lin *et al.* 2024, Zang *et al.* 2024, Cui *et al.* 2025). In contrast, Embed-638 was associated with suppression of immune effector pathways, including NK cell–mediated immunity and B cell proliferation (Fig. 5E and F and Table S9, available as supplementary data at *Bioinformatics Advances* online). The coordinated downregulation of these pathways indicates impaired cytotoxic and adaptive immune responses, consistent with an immunosuppressive tumor microenvironment. Ribosome biogenesis has been shown to limit immune responses, including NK cell activity, thus contributing to immune evasion in cancer (Bianco and Mohr 2019, Zang *et al.* 2024). These coordinated changes are characteristic of enhanced proliferative capacity coupled with immune suppression, a phenotype that has been associated with aggressive disease and adverse clinical outcomes in HNSC (Bianco and Mohr 2019, Wu *et al.* 2024, Zang *et al.* 2024).

Together, these results indicate that Embed-638 encodes biologically interpretable transcriptional programs that are prognostically relevant yet cancer-type specific. While elevated Embed-638 reflects immune activation accompanied by loss of neuronal differentiation in LGG, it captures a tumor-intrinsic growth–dominant and immunosuppressive state in HNSC, providing mechanistic context for its robust association with poor survival across malignancies.

## 4 Discussion

In this study, we systematically explored how foundation models can be leveraged to enhance cancer survival prediction. By using survival-associated gene expression as input, scFoundation-derived embeddings effectively retained the prognostic information inherent in gene expression data, and in some cancer types, these embeddings even provided greater robustness than individual genes (Tables S2 and S3, available as supplementary data at *Bioinformatics Advances* online). Consistent with prior studies, pan-cancer training improved both the accuracy and robustness of deep survival prediction models based on scFoundation embeddings. From a transfer learning perspective, this strategy can be viewed as a form of supervised fine-tuning across related cancer domains, enabling partial adaptation of pretrained representations to survival-specific objectives.

Our integrative model, EGSP, which combined embeddings, gene expression, and clinical features, achieved the best overall prognostic performance. Although scFoundation embeddings are derived from gene expression, we found that they could encode prognostic signals that extends beyond raw gene expression. These non-redundant risk embeddings were the key drivers of EGSP’s performance. Methodologically, EGSP adopts a feature fusion strategy through direct concatenation of complementary modalities, allowing the model to jointly leverage gene-level signals, latent embedding representations, and clinical covariates within a unified survival framework. Importantly, these non-redundant embeddings were primarily derived from the original pretrained scFoundation weights, whereas embeddings obtained from fine-tuning the last three transformer encoder layers through pan-cancer training (Embed-pan) exhibited greater redundancy (Fig. 3). This finding emerged from *post hoc* analyses rather than serving as a design assumption during model development. A possible explanation is that supervised pan-cancer training adjusts the weights toward minimizing Cox loss, potentially at the cost of higher-order gene interaction information captured during pretraining. This observation highlights a potential trade-off in transfer learning for survival analysis: while fine-tuning can improve task alignment, excessive adaptation may reduce the generality and diversity of pretrained representations.

The ability of scFoundation to extract non-redundant prognostic features suggests that its embeddings may serve as a new modality, complementing other data types to improve survival modeling. Biologically, such embeddings may summarize coordinated transcriptional programs, gene–gene interactions, or latent tumor states that are difficult to represent explicitly using individual gene features alone. Notably, integrating complementary embeddings from different models has already proven effective in tasks such as protein subcellular localization and function prediction (Hu *et al.* 2025), supporting the feasibility of this strategy. Future research should investigate whether other single-cell foundation models, such as GeneFormer (Theodoris *et al.* 2023), scGPT (Cui *et al.* 2024), or GenePT (Chen and Zou 2024), can also generate unique non-redundant risk embeddings. If so, integrating embeddings from multiple foundation models could provide an additional layer of complementary information for prognostic prediction. Such an approach would further extend the feature fusion paradigm explored in this study.

Although deep learning–based survival models often achieve high predictive accuracy, their translational utility is frequently limited by a lack of interpretability. To address this concern, we applied XAI techniques to interpret the EGSP model and systematically characterized the biological relevance of embedding features using SHAP and pathway-level analyses. This approach enabled us to move beyond black-box prediction and directly link model-derived embeddings to clinically and biologically meaningful processes. Our analysis identified Embed-638 as a robust prognostic feature with consistent importance at the pan-cancer level, and with stable and interpretable contributions in the LGG and HNSC cohorts. Importantly, SHAP-based interpretation revealed that the contribution of Embed-638 to survival prediction is context dependent, reflecting interactions with other molecular and clinical features rather than a simple marginal effect. Such behavior is expected in complex, nonlinear survival models and underscores the necessity of explainable methods to disentangle conditional feature effects. Functional characterization further demonstrated that Embed-638 captures distinct transcriptional programs in different tumor contexts. In LGG, elevated Embed-638 was associated with loss of neuronal differentiation signatures and increased immune-related activity, consistent with prior studies linking neuronal lineage features to favorable prognosis. In contrast, in HNSC, high Embed-638 reflected activation of tumor-intrinsic growth and translational programs coupled with suppression of antitumor immune responses, a phenotype characteristic of aggressive disease. These findings indicate that scFoundation-derived embeddings encode compact, biologically interpretable representations of tumor state rather than abstract latent variables. Together, these results highlight the translational potential of EGSP by demonstrating that its predictive features can be mechanistically interpreted, linked to known cancer biology, and decomposed at the individual-patient level. This framework provides a generalizable strategy for integrating foundation model–derived embeddings with explainable AI to enable both accurate and interpretable survival prediction in oncology.

An interesting observation from the SHAP analysis was that, in cancer types with limited sample sizes such as ESCA and UVM, EGSP achieved higher apparent performance while relying more heavily on clinical features, whereas EGSP-pan shifted predictive weight toward embedding features. Moreover, the wide confidence intervals of performance estimates in these cancers suggest that the observed improvements should be interpreted with caution. Together, these results illustrate how the trade-offs introduced by pan-cancer fine-tuning can manifest differently across cohorts, particularly in small-sample settings.

A limitation of this study is that model performance was primarily evaluated using discrimination-based metrics, including Harrell’s C-index, Uno’s C-index, and iAUC, which assess the correctness of risk ordering rather than the accuracy of absolute survival probability estimation or model calibration. While these metrics are appropriate for comparative prognostic modeling and risk stratification, accurate survival probability estimation and calibration are critical for clinical decision-making and translational application. Future work will therefore focus on extending the proposed framework to support calibrated survival prediction and more comprehensive evaluation of clinical utility.

Another limitation is that we only incorporated gene expression and clinical data. Considering that other modalities, such as WSI data, have been shown to substantially contribute to prognosis (Cheerla *et al.* 2019), further gains may be achieved by integrating foundation model–derived embeddings with diverse modalities. In parallel, foundation models have also been developed for other omics and data types, such as DNA methylation (Ying *et al.* 2024, de Lima Camillo *et al.* 2025), histopathology (Pai *et al.* 2024, Wang *et al.* 2024, Yang *et al.* 2025), and pathology reports (Labrak *et al.* 2024). In the foundation model era, integrating these complementary modalities holds significant potential to advance multi-omics foundation model–driven survival prediction and facilitate clinical translation.

## Supplementary Material

vbag076_Supplementary_Data

## Data Availability

No new data were generated or analyzed in support of this research. The complete implementation of EGSP, including training and inference pipelines and reproducible demo scripts, is publicly available at: https://github.com/weiliu123/EGSP.
