# Supplementary material for: Leveraging single-cell foundation models for accurate survival outcome prediction
Source: Bioinform Adv. 2026 Mar 16;6(1):vbag076. doi: 10.1093/bioadv/vbag076 (PMC13032892; doi:10.1093/bioadv/vbag076)
Supplement: vbag076_Supplementary_Data [file vbag076_supplementary_data.zip › Supplementary figures 20260308.docx]

## **Leveraging single-cell foundation models for accurate survival outcome prediction**

Wei Liu^1,*^, Qiang Wang^2^, Lin Long^3^, Wei Wang^1,*^

### **Supplementary Figures**


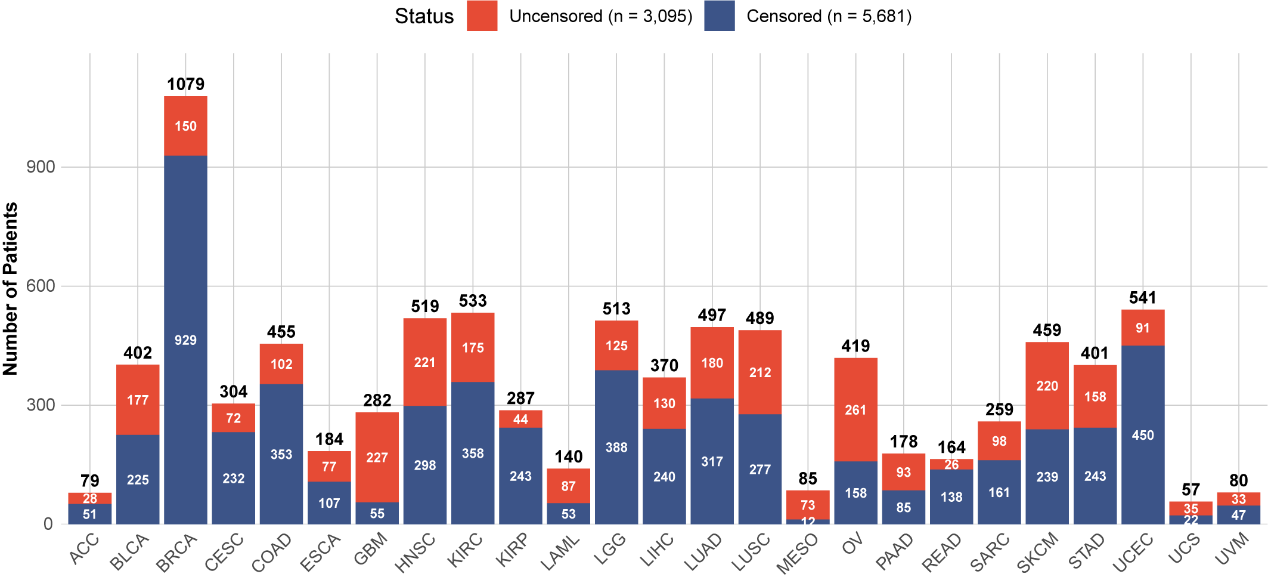


**Figure S1. Distribution of survival outcomes (uncensored vs. censored) across 25 cancer types selected for analysis in this study from the TCGA cohort.**


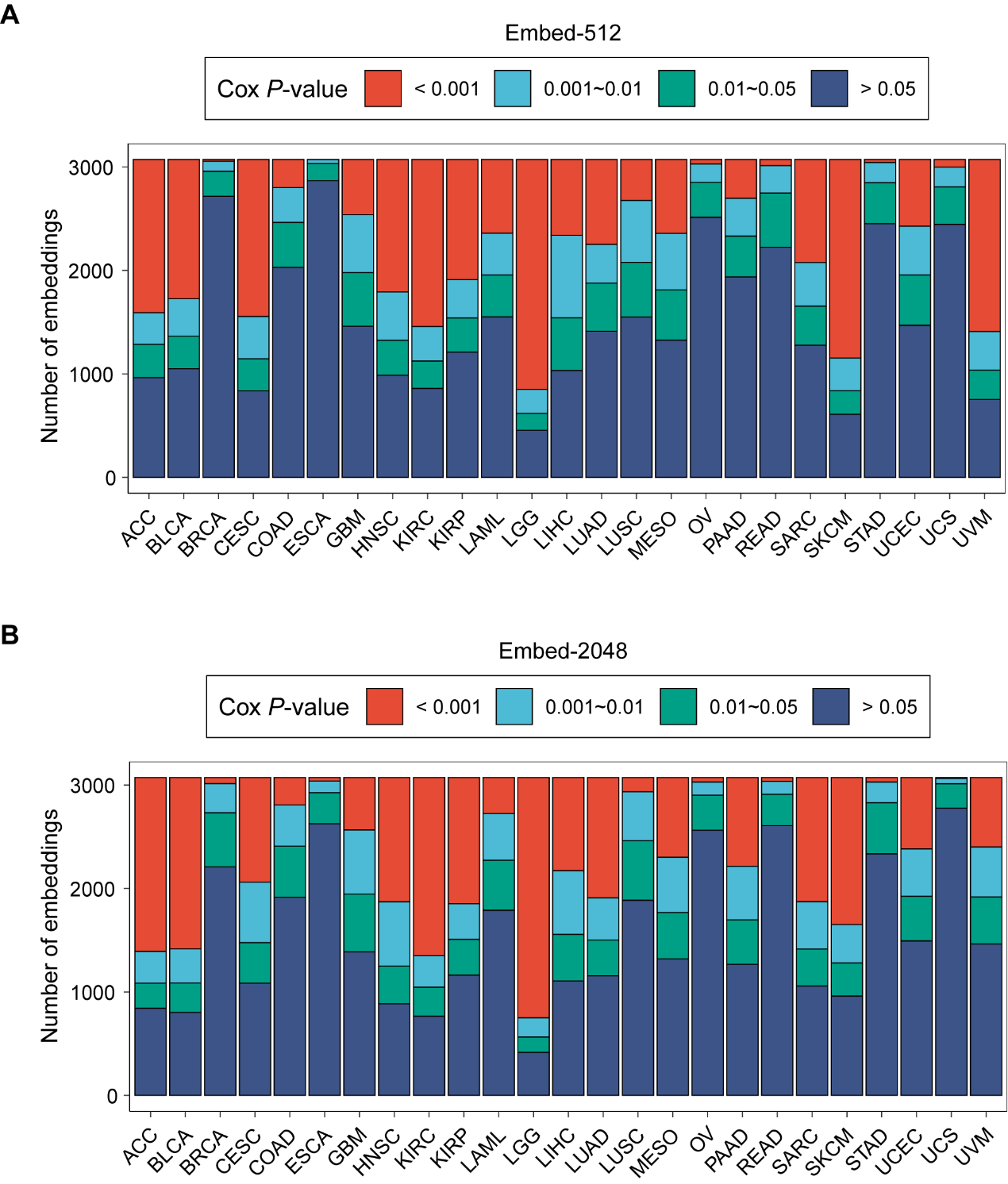


**Figure S2. Distribution of Cox *p*-values for the embeddings for each cancer type.** (**A**) Embed-512. (**B**) Embed-2048.


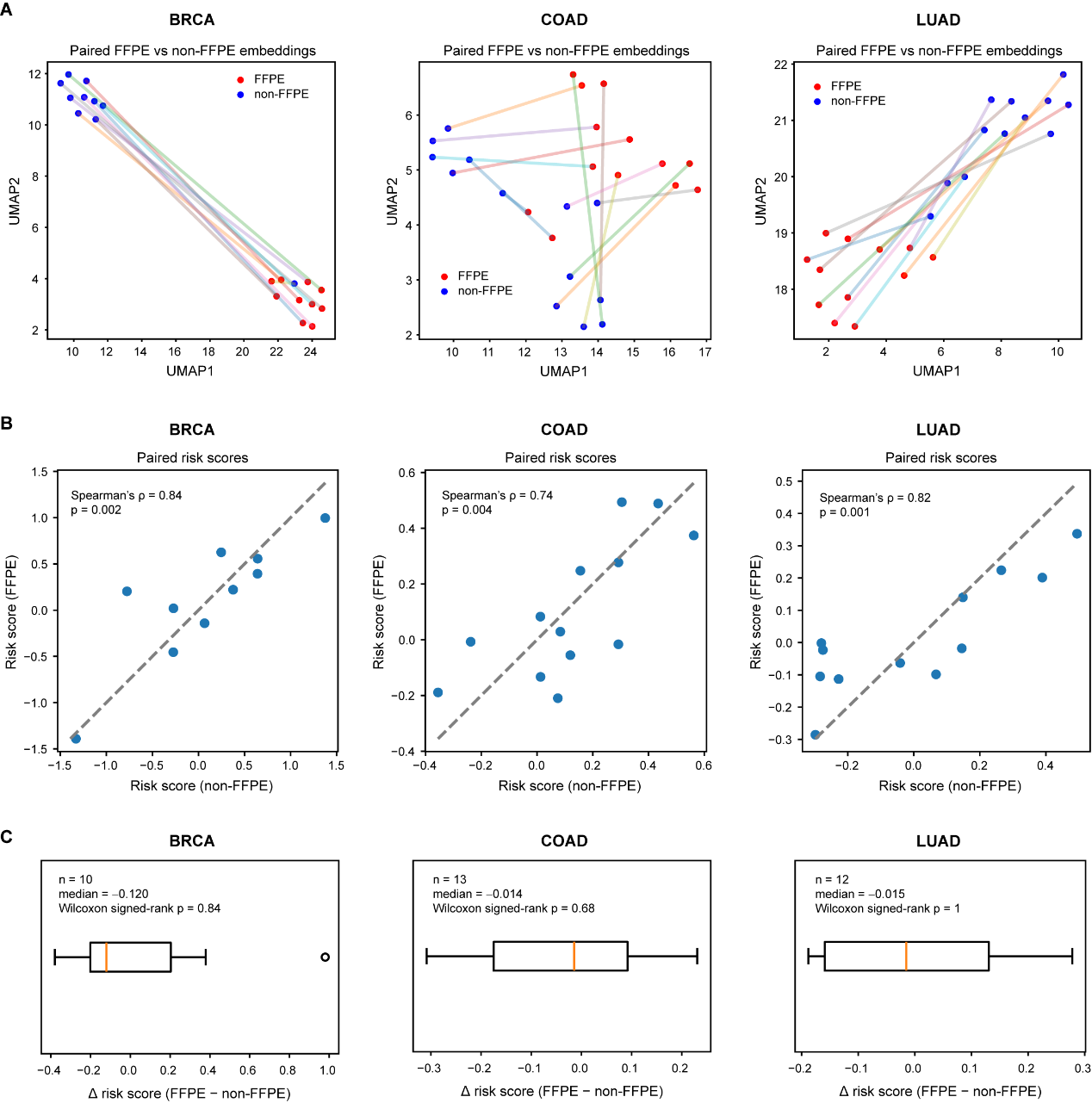


**Figure S3. Robustness of scFoundation-derived embeddings and risk prediction to FFPE processing.** (**A**) Paired FFPE and non-FFPE embeddings visualized by UMAP. For three representative cancer types (BRCA, COAD, and LUAD), UMAP projections of scFoundation-derived embeddings are shown for matched FFPE (red) and non-FFPE (blue) samples from the same patients. Lines connect paired specimens from each case, illustrating the relative displacement between FFPE and non-FFPE embeddings within the shared low-dimensional embedding space. (**B**) Concordance of EGSP-predicted risk scores between paired FFPE and non-FFPE samples. Scatter plots show predicted risk scores derived from FFPE samples versus those from matched non-FFPE samples for BRCA, COAD, and LUAD. The dashed line indicates the identity line (*y* = *x*). Spearman correlation coefficients and corresponding *p* values are shown in each panel. (**C**) Paired differences in EGSP-predicted risk scores between FFPE and non-FFPE samples. Risk differences were defined as Δrisk = FFPE − non-FFPE. Boxplots summarize the distribution of paired differences for each cancer type, with sample size, median, and Wilcoxon signed-rank *p* values indicated.


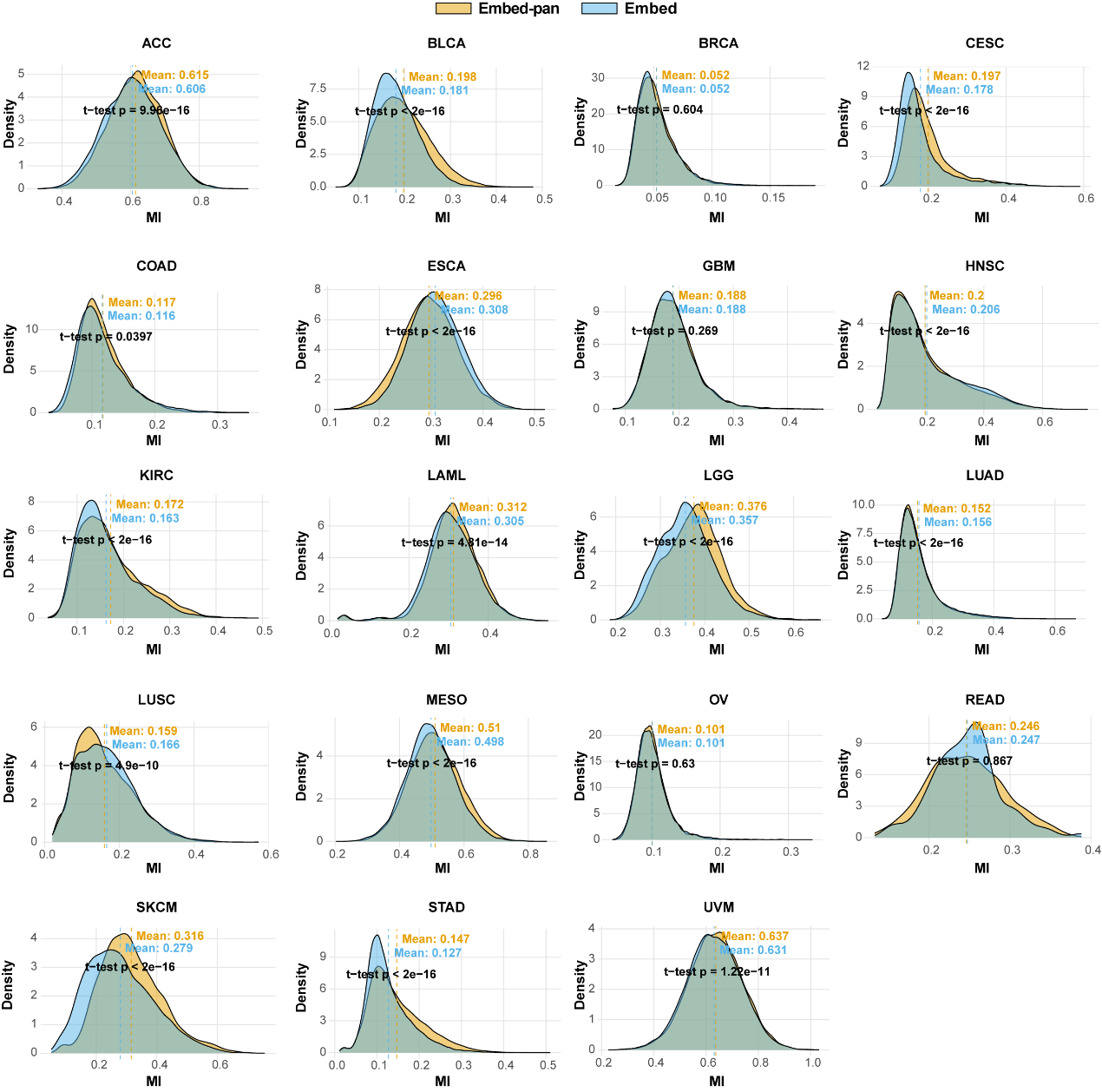


**Figure S4. Distribution of mutual information (MI) between embeddings and gene expression for 19 cancer types.** Embed: embeddings derived directly from pretrained scFoundation weights; Embed-pan: embeddings from scFoundation fine-tuned by unfreezing the last three transformer encoder layers in pan-cancer training.


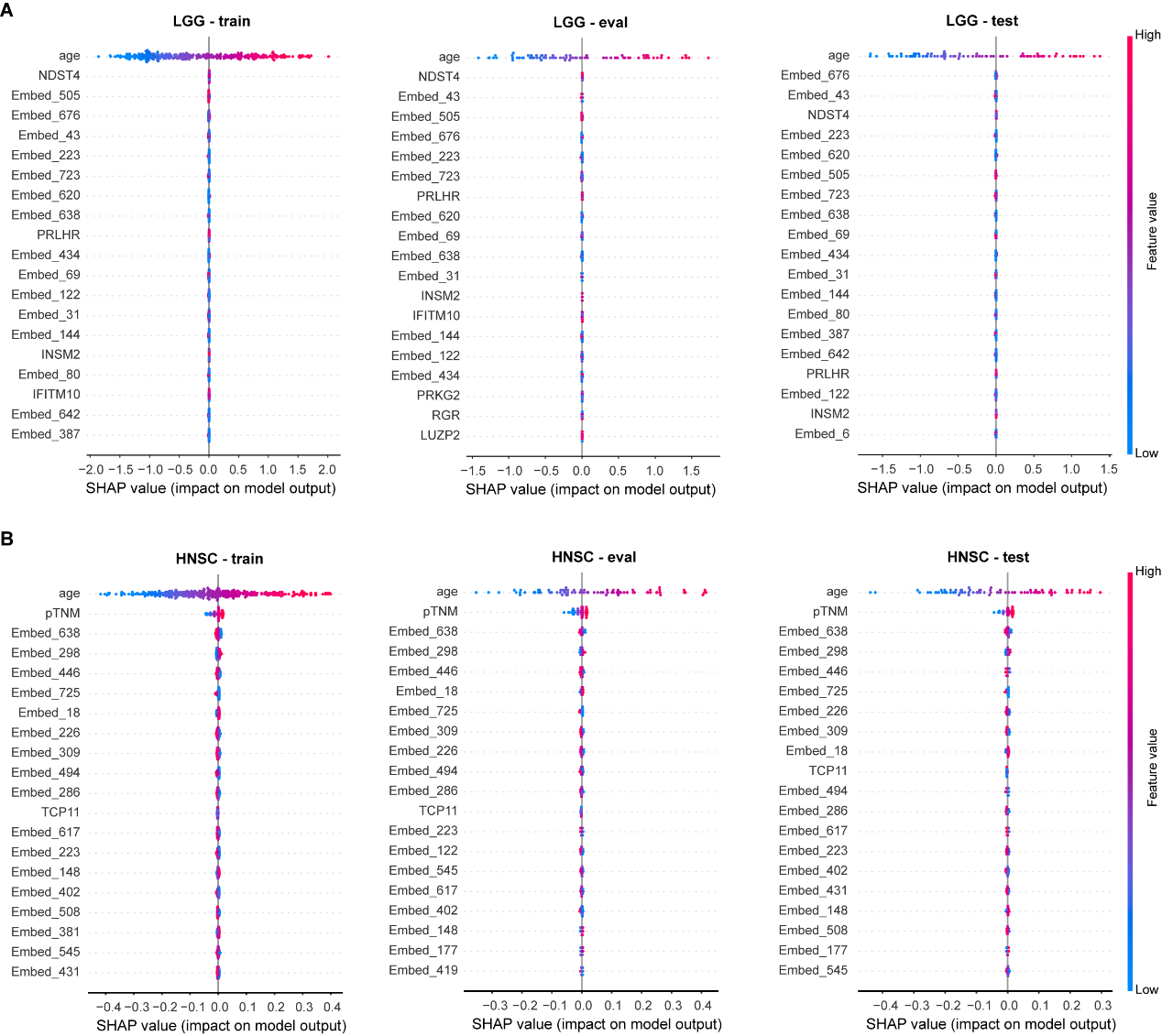


**Figure S5. SHAP summary plots for LGG and HNSC.** (**A**) SHAP summary plots showing the top 20 most influential features in the LGG cohort for the training (left), evaluation (middle), and test (right) datasets. Each dot represents an individual sample, with color indicating the feature value (red, high; blue, low). (**B**) SHAP summary plots for the HNSC cohort. Plot layout and visual conventions are the same as in panel **A**.


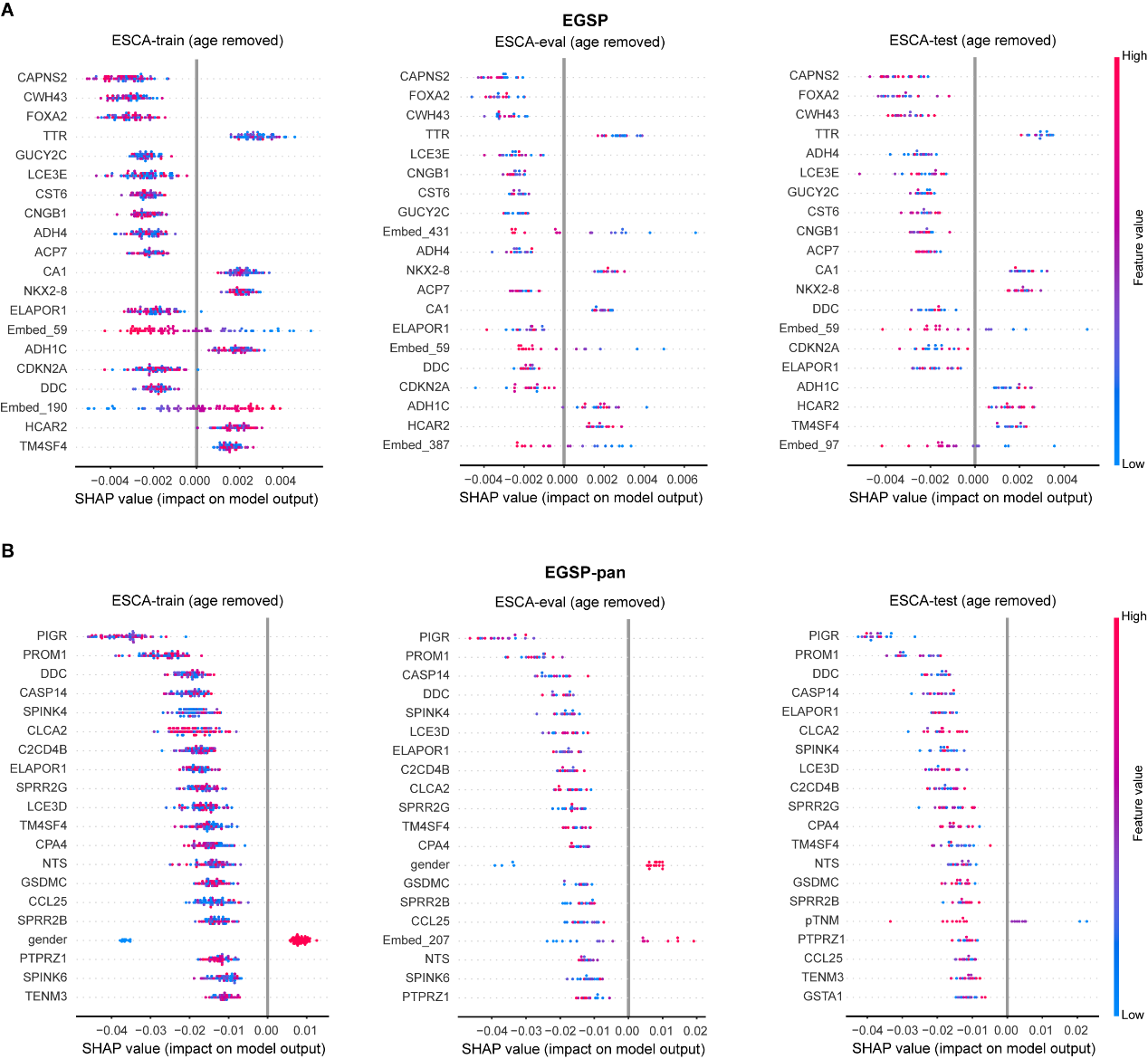


**Figure S6. Feature importance visualization for ESCC in EGSP and EGSP-pan models (age removed).** (**A**) SHAP summary plots showing the top 20 most influential features in the ESCC cohort for the training (left), evaluation (middle), and test (right) datasets using the EGSP model. Each dot represents a single sample, with color indicating the corresponding feature value (red, high; blue, low). For clarity, the age feature was excluded; the complete feature set is shown in the **Figure S7**. (**B**) SHAP summary plots for the EGSP-pan model, with the age feature removed for visualization. All other details are as described in panel **A**.


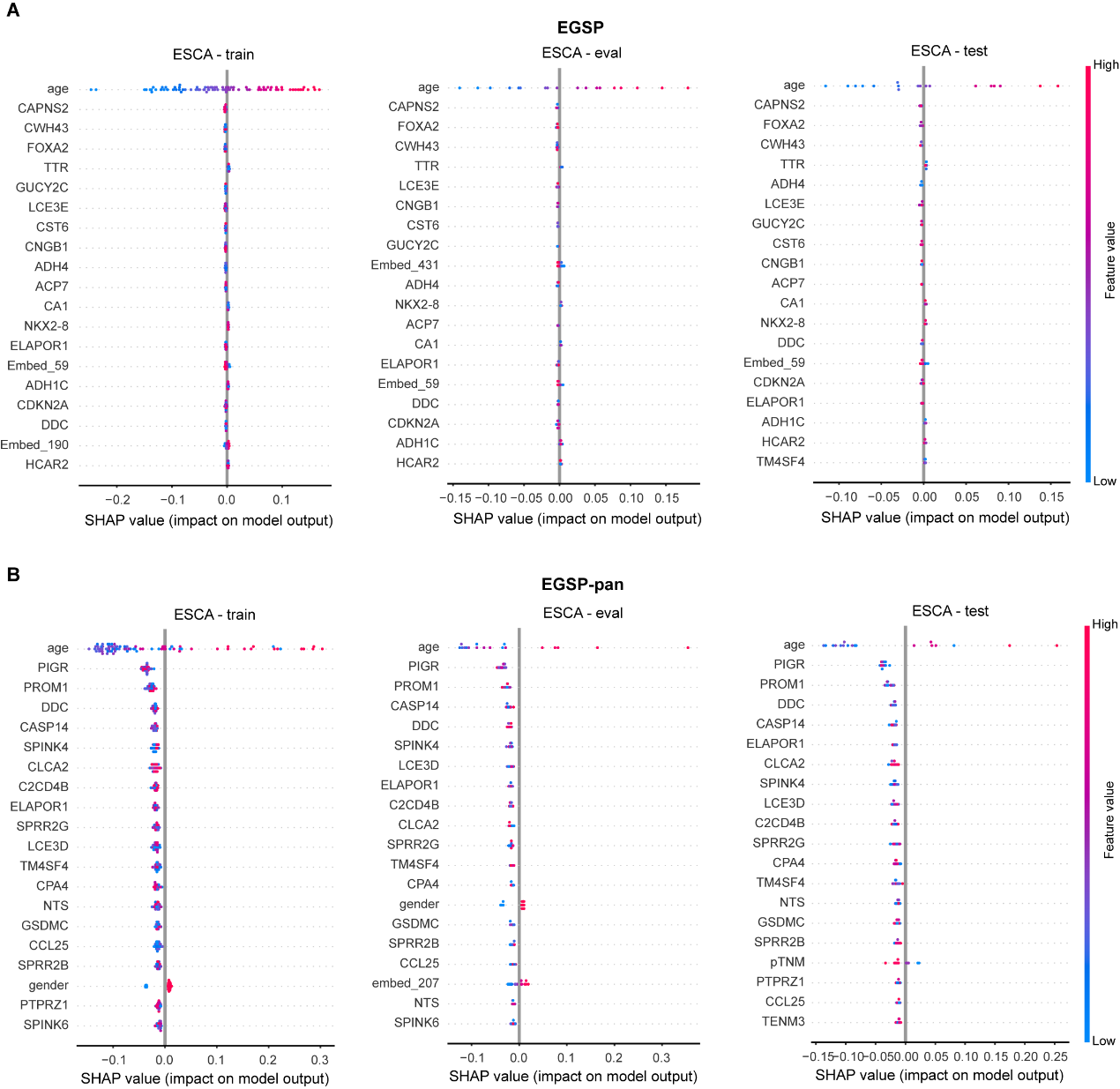


**Figure S7. Feature importance visualization for ESCC in EGSP and EGSP-pan models.** (**A**) SHAP summary plots showing the top 20 most influential features in the ESCC cohort for the training (left), evaluation (middle), and test (right) datasets using the EGSP model. Each dot represents a single sample, with color indicating the corresponding feature value (red, high; blue, low). (**B**) SHAP summary plots for the EGSP-pan model. All other details are as described in panel **A**.


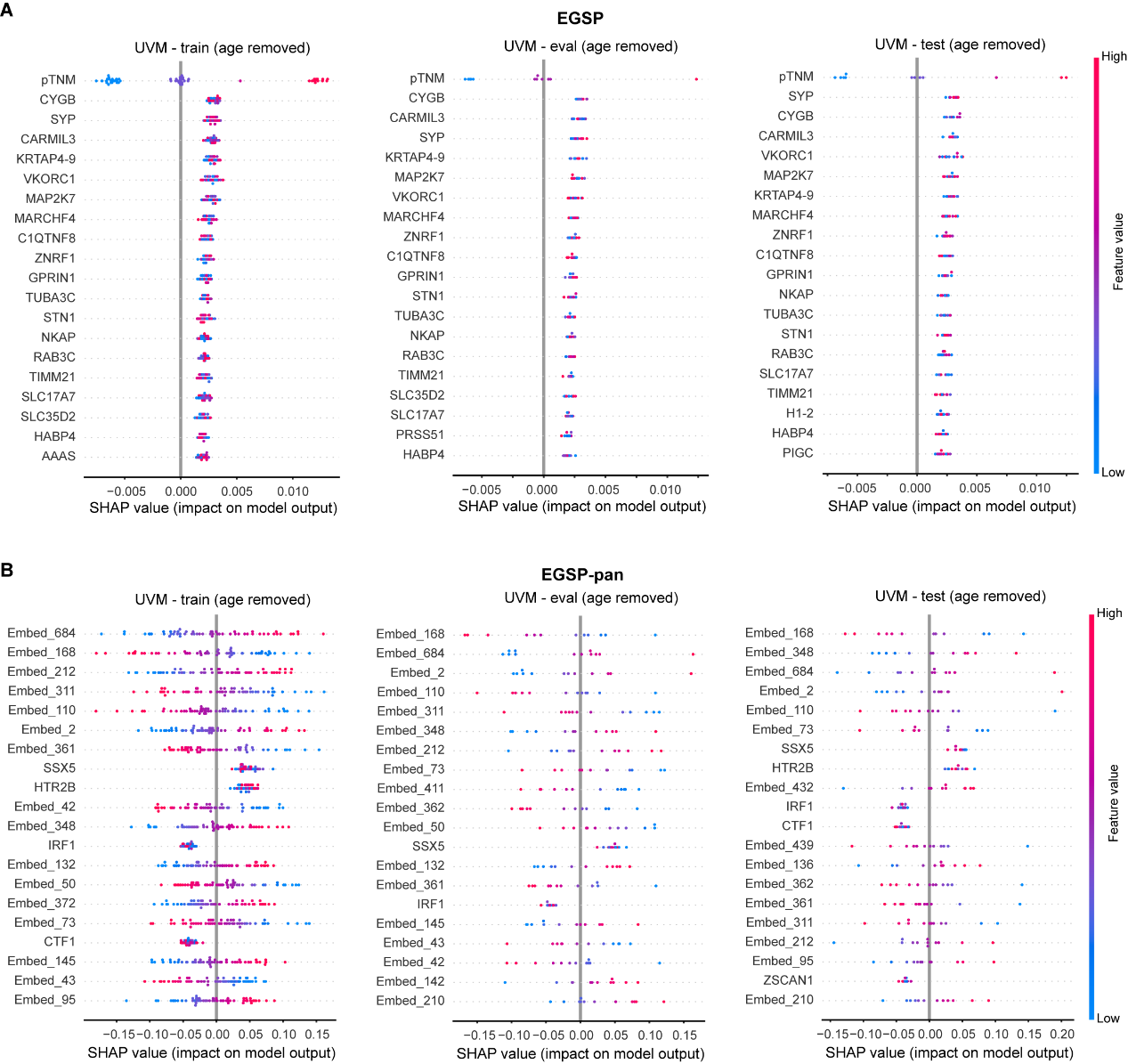


**Figure S8. Feature importance visualization for UVM in EGSP and EGSP-pan models (age removed).** (**A**) SHAP summary plots showing the top 20 most influential features in the UVM cohort for the training (left), evaluation (middle), and test (right) datasets using the EGSP model. Each dot represents a single sample, with color indicating the corresponding feature value (red, high; blue, low). For clarity, the age feature was excluded; the complete feature set is shown in the **Figure S9**. (**B**) SHAP summary plots for the EGSP-pan model, with the age feature removed for visualization. All other details are as described in panel **A**.


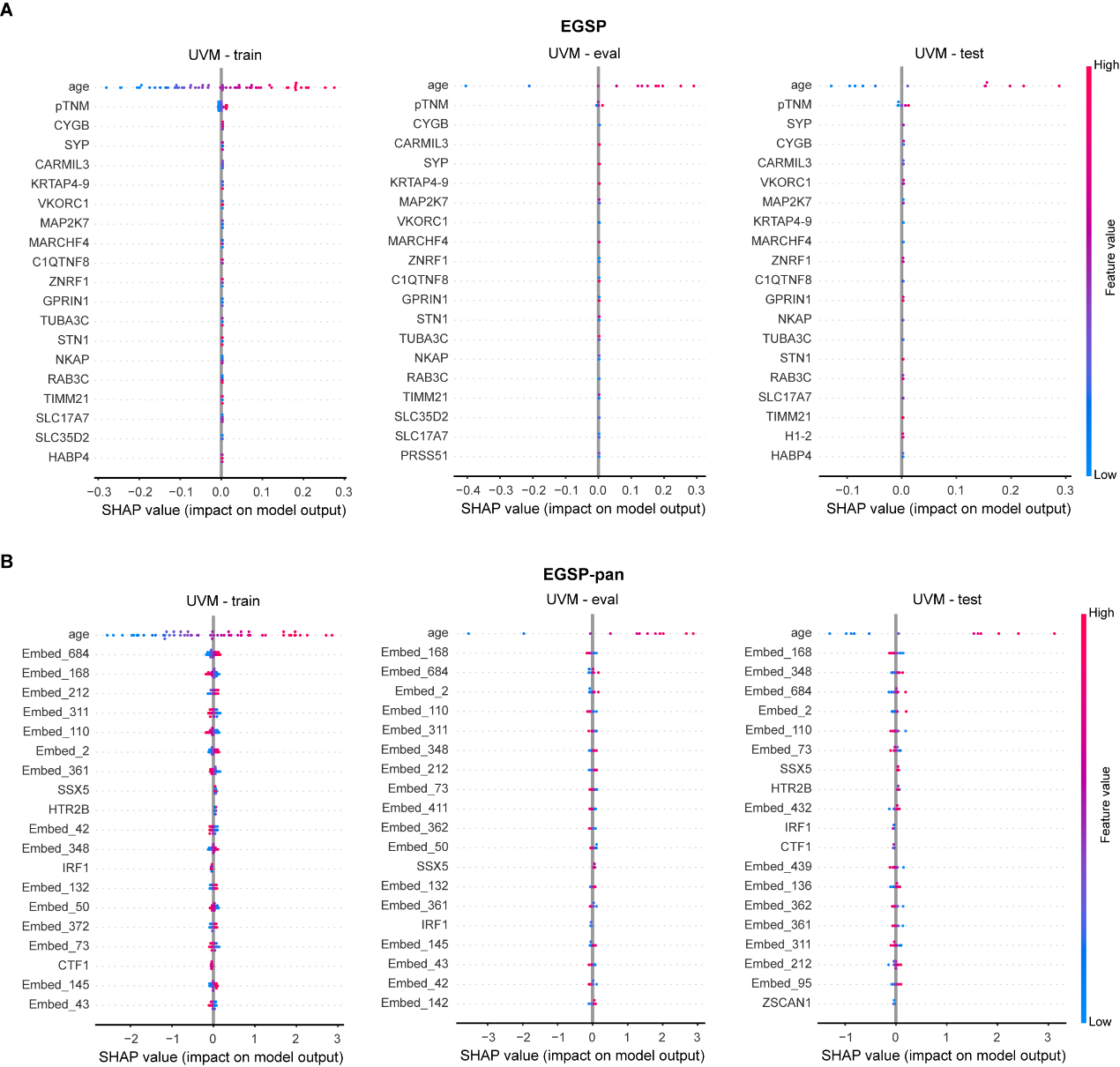


**Figure S9. Feature importance visualization for UVM in EGSP and EGSP-pan models.** (**A**) SHAP summary plots showing the top 20 most influential features in the UVM cohort for the training (left), evaluation (middle), and test (right) datasets using the EGSP model. Each dot represents a single sample, with color indicating the corresponding feature value (red, high; blue, low). (**B**) SHAP summary plots for the EGSP-pan model. All other details are as described in panel **A**.


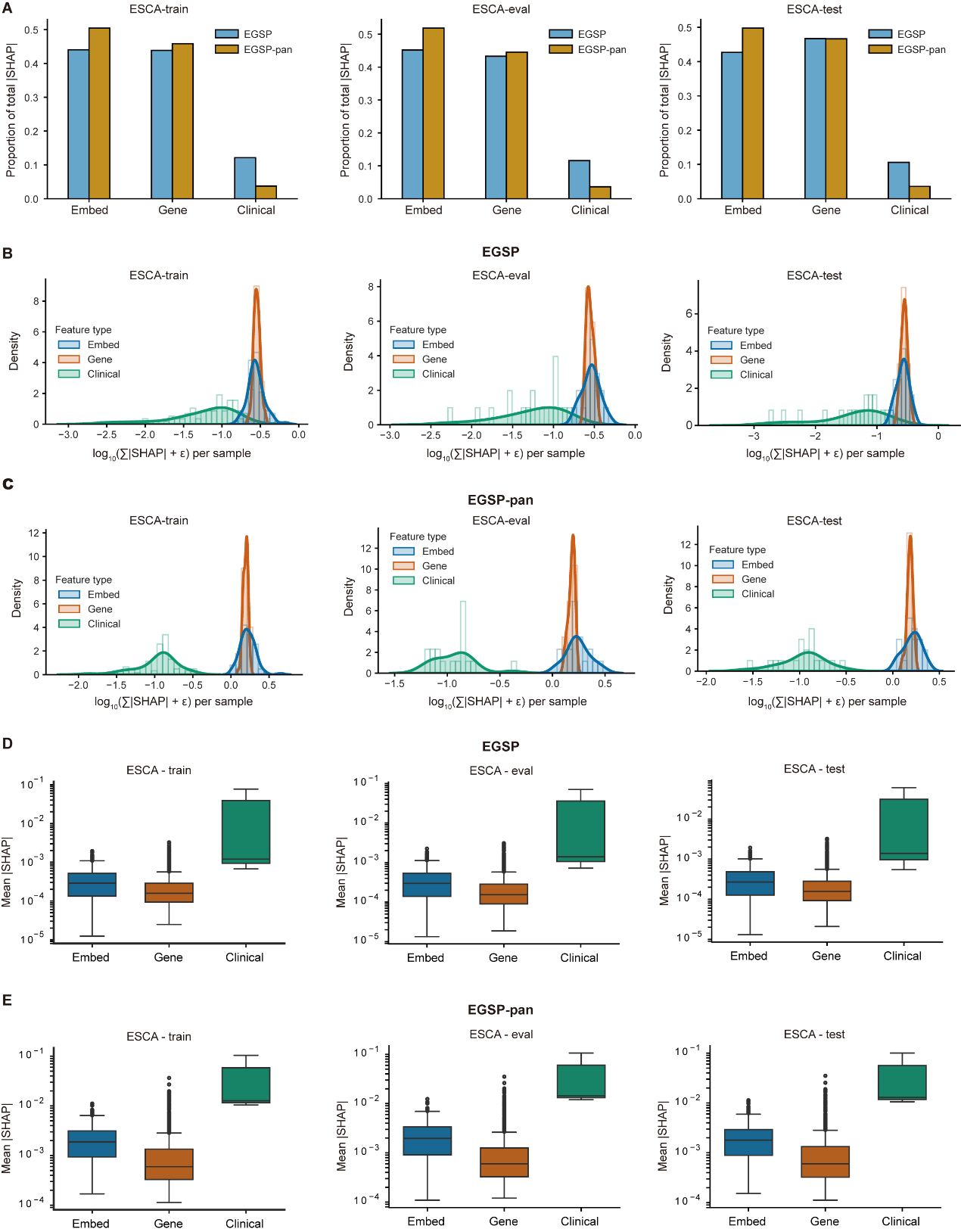


**Figure S10. SHAP value analysis of embedding, gene, and clinical features in the ESCA cohort.** (**A**) Bar plots showing the proportion of total absolute SHAP values attributed to embedding, gene, and clinical features in the training (left), evaluation (middle), and test (right) sets of the ESCA cohort. Results are presented for both EGSP (blue) and EGSP-pan (yellow) models. (**B**, **C**) Density distributions of per-sample SHAP attributions across embedding, gene, and clinical features for the ESCA cohort using the EGSP (**B**) and EGSP-pan (**C**) models. For each sample, SHAP values were summed within each feature category, log-transformed, and normalized within feature type to enable comparison of distributional shapes across sets. (**D**, **E**) Boxplots showing the mean absolute SHAP values of embedding, gene, and clinical features in the ESCA cohort for the training (left), evaluation (middle), and test (right) sets using the EGSP (**D**) and EGSP-pan (**E**) models. The y-axis is displayed on a logarithmic scale. Embed: embedding feature; Gene: gene feature; Clinical: clinical feature.


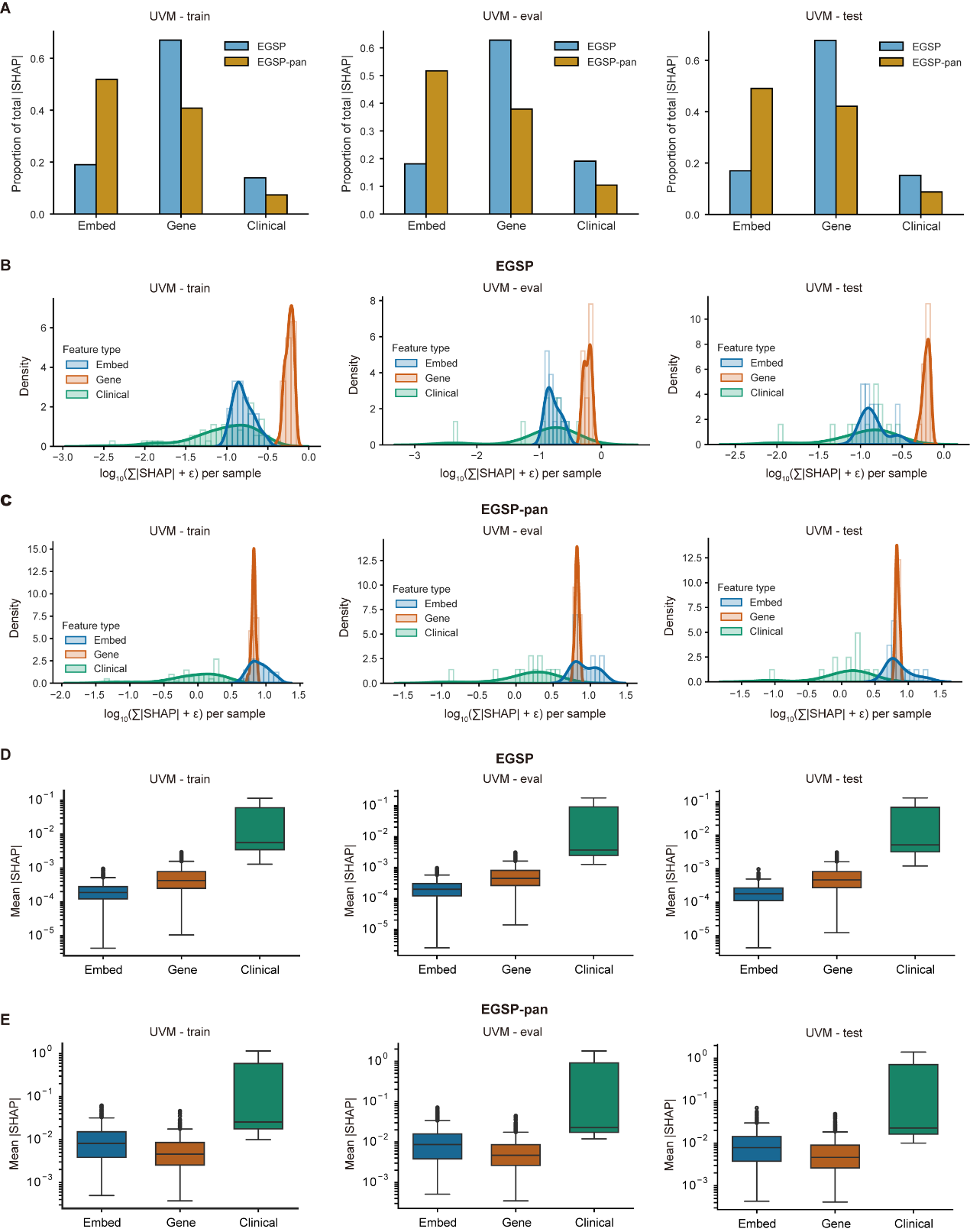


**Figure S11. SHAP value analysis of embedding, gene, and clinical features in the UVM cohort.** (**A**) Bar plots showing the proportion of total absolute SHAP values attributed to embedding, gene, and clinical features in the training (left), evaluation (middle), and test (right) sets of the UVM cohort. Results are presented for both EGSP (blue) and EGSP-pan (yellow) models. (**B**, **C**) Density distributions of per-sample SHAP attributions across embedding, gene, and clinical features for the UVM cohort using the EGSP (**B**) and EGSP-pan (**C**) models. For each sample, SHAP values were summed within each feature category, log-transformed, and normalized within feature type to enable comparison of distributional shapes across sets. (**D**, **E**) Boxplots showing the mean absolute SHAP values of embedding, gene, and clinical features in the UVM cohort for the training (left), evaluation (middle), and test (right) sets using the EGSP (**D**) and EGSP-pan (**E**) models. The y-axis is displayed on a logarithmic scale. Embed: embedding feature; Gene: gene feature; Clinical: clinical feature.
