# Supplementary material for: Leveraging single-cell foundation models for accurate survival outcome prediction
Source: Bioinform Adv. 2026 Mar 16;6(1):vbag076. doi: 10.1093/bioadv/vbag076 (PMC13032892; doi:10.1093/bioadv/vbag076)
Supplement: vbag076_Supplementary_Data [file vbag076_supplementary_data.zip › Supplementary Tables 20260308.docx]

## **Leveraging single-cell foundation models for accurate survival outcome prediction**

Wei Liu^1,*^, Qiang Wang^2^, Lin Long^3^, Wei Wang^1,*^

### **Supplementary Tables S1–S7**

**Table S1. Computational cost of EGSP under different experimental settings.** Wall-clock time and peak GPU memory usage were measured on a single workstation equipped with an NVIDIA RTX 3090 GPU. EGSP refers to the embedding-based configuration using precomputed embeddings, while EGSP_End2End denotes the end-to-end configuration with internal embedding extraction via scFoundation. In all experiments, mRNA expression features were represented using 1,024 selected genes, and models were trained with a per-device training batch size of 24.

| Model | Input type | #Samples | #Epochs | Wall-clock time (min) | Peak GPU memory (GB) |
| --- | --- | --- | --- | --- | --- |
| EGSP | Embeddings + mRNA + Clinical | 100 | - | 0.0015 | <0.01 |
| EGSP | Embeddings + mRNA + Clinical | 100 | 10 | 0.020 | 0.07 |
| EGSP_End2End | mRNA + Clinical | 100 | 10 | 0.54 | 3.47 |
| EGSP_End2End | mRNA + Clinical | ~7,200 | 10 | 42.9 | 5.39 |

**Table S2. Prognostic performance of scFoundation embeddings with varying input gene numbers evaluated by the Ridge-Cox model.**

| Cancer | mRNA | Embed-512 | Embed-1024 | Embed-2048 |
| --- | --- | --- | --- | --- |
| ACC | 0.888 | 0.916 | **0.929** | 0.898 |
| BLCA | 0.695 | **0.680** | 0.672 | 0.672 |
| BRCA | 0.699 | **0.613** | 0.579 | 0.586 |
| CESC | 0.767 | **0.722** | 0.716 | 0.682 |
| COAD | 0.612 | 0.613 | 0.612 | **0.639** |
| ESCA | 0.765 | 0.577 | **0.633** | 0.614 |
| GBM | 0.608 | 0.577 | **0.603** | 0.561 |
| HNSC | 0.688 | 0.665 | **0.672** | 0.669 |
| KIRC | 0.679 | 0.675 | **0.669** | 0.663 |
| KIRP | 0.817 | 0.719 | 0.758 | **0.782** |
| LAML | 0.680 | **0.701** | 0.664 | 0.657 |
| LGG | 0.806 | 0.756 | 0.773 | **0.782** |
| LIHC | 0.685 | 0.655 | 0.671 | **0.672** |
| LUAD | 0.624 | 0.652 | 0.651 | **0.657** |
| LUSC | 0.597 | **0.623** | 0.620 | 0.600 |
| MESO | 0.635 | 0.691 | **0.702** | 0.671 |
| OV | 0.608 | **0.574** | 0.565 | 0.568 |
| PAAD | 0.544 | **0.697** | 0.633 | 0.672 |
| READ | 0.615 | 0.628 | **0.668** | 0.627 |
| SARC | 0.697 | **0.722** | 0.711 | 0.713 |
| SKCM | 0.657 | **0.627** | 0.618 | 0.601 |
| STAD | 0.623 | 0.566 | **0.576** | 0.555 |
| UCEC | 0.642 | 0.694 | 0.690 | **0.709** |
| UCS | 0.645 | **0.772** | 0.761 | 0.624 |
| UVM | 0.885 | 0.839 | **0.862** | 0.805 |
| Mean | 0.686±0.089 | 0.678±0.083 | **0.680±0.086** | 0.667±0.081 |

*Note:* *mRNA* indicates Cox models based on gene expression features. Embed-512, Embed-1024, and Embed-2048 denote embeddings derived from the top 512, 1024, and 2048 survival-associated genes, respectively. For each cancer type, the best-performing embedding (among Embed-512, Embed-1024, and Embed-2048) is highlighted in bold.

**Table S3. Prognostic performance of scFoundation embeddings with varying input gene numbers evaluated by the Lasso-Cox model.**

| Cancer | mRNA | Embed-512 | Embed-1024 | Embed-2048 |
| --- | --- | --- | --- | --- |
| ACC | 0.872 | **0.914** | 0.853 | 0.912 |
| BLCA | 0.613 | 0.682 | 0.660 | **0.685** |
| BRCA | 0.556 | 0.588 | **0.610** | 0.608 |
| CESC | 0.587 | 0.700 | **0.721** | 0.681 |
| COAD | 0.566 | 0.610 | 0.622 | **0.661** |
| ESCA | 0.506 | 0.501 | **0.668** | 0.608 |
| GBM | 0.592 | **0.576** | 0.566 | 0.555 |
| HNSC | 0.672 | 0.661 | **0.673** | 0.669 |
| KIRC | 0.648 | 0.658 | 0.649 | **0.669** |
| KIRP | 0.702 | 0.716 | **0.789** | 0.722 |
| LAML | 0.640 | **0.704** | 0.653 | 0.676 |
| LGG | 0.787 | 0.745 | **0.772** | 0.764 |
| LIHC | 0.649 | **0.676** | 0.641 | 0.653 |
| LUAD | 0.596 | 0.654 | 0.639 | **0.679** |
| LUSC | - | **0.613** | 0.599 | 0.612 |
| MESO | 0.609 | 0.677 | **0.687** | 0.660 |
| OV | 0.502 | 0.567 | 0.532 | **0.589** |
| PAAD | 0.527 | **0.647** | 0.625 | 0.614 |
| READ | 0.417 | 0.690 | 0.676 | **0.747** |
| SARC | 0.540 | 0.718 | **0.767** | 0.726 |
| SKCM | 0.642 | 0.600 | **0.618** | 0.584 |
| STAD | 0.550 | 0.531 | 0.531 | **0.551** |
| UCEC | 0.529 | 0.647 | 0.666 | **0.731** |
| UCS | 0.334 | **0.751** | 0.744 | 0.451 |
| UVM | 0.840 | **0.850** | 0.787 | 0.787 |
| Mean | 0.603±0.120 | 0.667±0.091 | **0.670±0.081** | 0.664±0.091 |

*Note:* *mRNA* indicates Cox models based on gene expression features. Embed-512, Embed-1024, and Embed-2048 denote embeddings derived from the top 512, 1024, and 2048 survival-associated genes, respectively. For each cancer type, the best-performing embedding is highlighted in bold.

**Table S4. Performance comparison of different combinations of data modalities and training strategies on 25 cancer types using iAUC.**

| Cancer | N (patients) | Censoring rate (%) | Embed | Embed+Clin | | Embed-pan+Clin | | mRNA+Clin | Embed-pan+mRNA+Clin | | | | Embed+mRNA+Clin | | | |
| --- | --- | --- | --- | --- | --- | --- | --- | --- | --- | --- | --- | --- | --- | --- | --- | --- |
|  |  |  |  |  |  |  |  |  | Lasso-Cox | Ridge-Cox | | EGSP-pan | Lasso-Cox | Ridge-Cox | | EGSP |
| ACC | 79 | 64.6 | 0.595 | | 0.604 | 0.874 | 0.843 | | 0.921 | 0.873 | 0.835 | | 0.958 | 0.833 | **0.981** | |
| BLCA | 402 | 56.0 | 0.639 | | 0.676 | 0.685 | **0.743** | | 0.720 | 0.695 | 0.715 | | 0.702 | 0.699 | 0.717 | |
| BRCA | 1079 | 68.1 | 0.501 | | 0.678 | 0.640 | 0.732 | | 0.719 | 0.657 | 0.707 | | 0.701 | 0.614 | **0.782** | |
| CESC | 304 | 76.3 | 0.778 | | 0.765 | 0.753 | 0.755 | | 0.384 | 0.567 | **0.789** | | 0.500 | 0.503 | 0.788 | |
| COAD | 455 | 77.6 | 0.666 | | 0.655 | 0.669 | 0.688 | | **0.739** | 0.705 | 0.522 | | 0.730 | 0.682 | 0.716 | |
| ESCA | 184 | 58.2 | 0.584 | | 0.698 | 0.743 | 0.702 | | 0.634 | 0.435 | 0.693 | | 0.720 | 0.511 | **0.952** | |
| GBM | 282 | 19.5 | 0.554 | | **0.688** | 0.658 | 0.652 | | 0.618 | 0.471 | 0.654 | | 0.683 | 0.486 | 0.645 | |
| HNSC | 519 | 57.4 | **0.729** | | 0.726 | 0.666 | 0.715 | | 0.483 | 0.414 | 0.694 | | 0.524 | 0.518 | 0.706 | |
| KIRC | 533 | 67.2 | 0.714 | | 0.691 | 0.706 | 0.784 | | 0.796 | 0.774 | 0.753 | | 0.794 | 0.786 | **0.837** | |
| KIRP | 287 | 84.7 | 0.894 | | 0.879 | 0.898 | 0.900 | | 0.522 | 0.729 | 0.846 | | 0.424 | 0.631 | **0.941** | |
| LAML | 140 | 37.9 | 0.706 | | 0.831 | 0.808 | 0.874 | | 0.253 | 0.416 | 0.904 | | 0.491 | 0.677 | **0.882** | |
| LGG | 513 | 75.6 | 0.843 | | 0.767 | 0.814 | 0.816 | | 0.154 | 0.487 | 0.852 | | 0.320 | 0.731 | **0.869** | |
| LIHC | 370 | 64.9 | 0.494 | | 0.525 | 0.542 | 0.618 | | 0.601 | 0.682 | 0.576 | | 0.663 | **0.698** | 0.668 | |
| LUAD | 497 | 63.8 | 0.737 | | 0.655 | 0.701 | 0.810 | | 0.689 | 0.686 | **0.860** | | 0.750 | 0.799 | 0.799 | |
| LUSC | 489 | 56.6 | 0.610 | | 0.632 | **0.697** | 0.652 | | 0.564 | 0.516 | 0.676 | | 0.555 | 0.503 | 0.669 | |
| MESO | 85 | 14.1 | 0.657 | | 0.781 | 0.681 | **0.849** | | 0.321 | 0.790 | 0.793 | | 0.201 | 0.780 | 0.844 | |
| OV | 419 | 37.7 | 0.433 | | 0.642 | 0.589 | 0.627 | | 0.490 | 0.544 | 0.706 | | 0.502 | 0.520 | **0.714** | |
| PAAD | 178 | 47.8 | 0.433 | | 0.405 | 0.577 | 0.456 | | 0.612 | 0.558 | **0.636** | | 0.609 | 0.517 | 0.576 | |
| READ | 164 | 84.1 | 0.651 | | 0.665 | **0.777** | 0.538 | | 0.395 | 0.507 | 0.642 | | 0.402 | 0.468 | 0.642 | |
| SARC | 259 | 62.2 | 0.688 | | 0.467 | 0.498 | 0.653 | | 0.500 | 0.488 | **0.751** | | 0.510 | 0.405 | 0.677 | |
| SKCM | 459 | 52.1 | 0.677 | | 0.755 | **0.770** | 0.746 | | 0.506 | 0.326 | 0.745 | | 0.525 | 0.361 | 0.759 | |
| STAD | 401 | 60.6 | 0.422 | | **0.690** | 0.599 | 0.629 | | 0.665 | 0.530 | 0.676 | | 0.712 | 0.536 | 0.630 | |
| UCEC | 541 | 83.2 | 0.650 | | 0.477 | 0.536 | 0.672 | | **0.730** | 0.696 | 0.488 | | 0.722 | 0.588 | 0.704 | |
| UCS | 57 | 38.6 | 0.254 | | 0.619 | 0.937 | 0.250 | | 0.210 | 0.665 | **0.987** | | 0.283 | 0.608 | 0.767 | |
| UVM | 80 | 58.8 | 0.664 | | 0.770 | 0.802 | 0.740 | | 0.817 | 0.650 | 0.786 | | 0.806 | 0.650 | **1.00** | |
| Mean±SD | - | - | 0.623±0.142 | | 0.670±0.112 | 0.705±0.114 | 0.698±0.140 | | 0.562±0.196 | 0.594±0.137 | 0.731±0.116 | | 0.592±0.180 | 0.604±0.128 | **0.771**±**0.117** | |

Note: For each cancer type, the best result is shown in bold. Embed: embeddings derived directly from pretrained scFoundation weights; Embed-pan: embeddings from scFoundation fine-tuned by unfreezing the last three encoder layers in pan-cancer training; Clin, clinical data; mRNA, mRNA expression data; EGSP-pan, model integrating Embed-pan, gene expression, and clinical features; EGSP, model integrating Embed, gene expression, and clinical features.**Table S5. Performance comparison of different combinations of data modalities and training strategies on 25 cancer types using Uno's C-index (95% CI).**

| Cancer | N (patients) | Censoring rate (%) | Embed | Embed+Clin | | Embed-pan+Clin | | mRNA+Clin | Embed-pan+mRNA+Clin | | | | Embed+mRNA+Clin | | | |
| --- | --- | --- | --- | --- | --- | --- | --- | --- | --- | --- | --- | --- | --- | --- | --- | --- |
|  |  |  |  |  |  |  |  |  | Lasso-Cox | Ridge-Cox | | EGSP-pan | Lasso-Cox | Ridge-Cox | | EGSP |
| ACC | 79 | 64.6 | 0.59 (NA)^a^ | | 0.69 (NA) | **0.89 (NA)** | 0.65 (NA) | | 0.82 (NA) | 0.75 (NA) | 0.70 (NA) | | 0.82 (NA) | 0.82 (NA) | 0.76 (NA) | |
| BLCA | 402 | 56.0 | 0.64 (0.52-0.75) | | 0.65 (0.53-0.75) | 0.68 (0.57-0.78) | **0.70 (0.59-0.80)** | | 0.67 (0.55-0.78) | 0.67 (0.56-0.78) | 0.69 (0.57-0.80) | | 0.66 (0.55-0.77) | 0.69 (0.58-0.80) | 0.68 (0.57-0.79) | |
| BRCA | 1079 | 68.1 | 0.52 (0.38-0.65) | | 0.63 (0.50-0.75) | 0.62 (0.48-0.75) | 0.73 (0.61-0.82) | | 0.58 (0.41-0.75) | 0.59 (0.43-0.75) | 0.69 (0.56-0.80) | | 0.58 (0.40-0.75) | 0.58 (0.39-0.76) | **0.75 (0.64-0.84)** | |
| CESC | 304 | 76.3 | **0.63 (0.43-0.82)** | | 0.61 (0.43-0.80) | 0.58 (0.38-0.79) | 0.57 (0.35-0.79) | | 0.40 (0.21-0.61) | 0.55 (0.36-0.76) | 0.59 (0.38-0.81) | | 0.43 (0.25-0.64) | 0.51 (0.33-0.74) | 0.60 (0.40-0.81) | |
| COAD | 455 | 77.6 | 0.59 (0.40-0.77) | | 0.60 (0.40-0.80) | 0.56 (0.37-0.78) | 0.61 (0.42-0.81) | | 0.74 (0.54-0.90) | 0.74 (0.47-0.92) | 0.59 (0.42-0.77) | | 0.69 (0.49-0.89) | **0.74 (0.50-0.91)** | 0.64 (0.36-0.88) | |
| ESCA | 184 | 58.2 | 0.46 (0.22-0.69) | | 0.56 (0.32-0.80) | 0.61 (0.38-0.81) | 0.46 (0.24-0.73) | | 0.50 (0.22-0.84) | 0.27 (0.08-0.58) | 0.59 (0.37-0.80) | | 0.63 (0.36-0.90) | 0.34 (0.14-0.62) | **0.84 (0.12-0.99)** | |
| GBM | 282 | 19.5 | 0.58 (0.43-0.71) | | **0.65 (0.50-0.78)** | 0.64 (0.50-0.77) | 0.66 (0.52-0.79) | | 0.57 (0.44-0.69) | 0.44 (0.31-0.58) | 0.61 (0.47-0.73) | | 0.62 (0.50-0.73) | 0.45 (0.32-0.58) | 0.62 (0.48-0.75) | |
| HNSC | 519 | 57.4 | 0.54 (0.40-0.68) | | 0.51 (0.36-0.65) | 0.50 (0.35-0.65) | 0.52 (0.38-0.68) | | 0.52 (0.39-0.64) | 0.44 (0.30-0.58) | 0.56 (0.42-0.71) | | 0.56 (0.43-0.67) | 0.53 (0.38-0.68) | **0.60 (0.47-0.72)** | |
| KIRC | 533 | 67.2 | 0.70 (0.57-0.81) | | 0.77 (0.63-0.88) | 0.75 (0.62-0.85) | 0.78 (0.67-0.87) | | 0.81 (0.70-0.90) | 0.77 (0.66-0.87) | 0.74 (0.63-0.85) | | 0.81 (0.69-0.90) | 0.77 (0.66-0.87) | **0.87 (0.75-0.94)** | |
| KIRP | 287 | 84.7 | 0.84 (0.57-1.00) | | 0.83 (0.58-0.98) | 0.82 (0.57-0.98) | **0.91 (0.80-0.98)** | | 0.61 (0.31-0.87) | 0.80 (0.52-1.00) | 0.71 (0.39-0.96) | | 0.56 (0.26-0.83) | 0.69 (0.37-0.97) | 0.87 (0.72-0.99) | |
| LAML | 140 | 37.9 | 0.66 (0.46-0.83) | | 0.69 (0.56-0.82) | 0.71 (0.56-0.84) | 0.78 (0.67-0.90) | | 0.35 (0.20-0.50) | 0.47 (0.28-0.67) | 0.76 (0.65-0.88) | | 0.46 (0.27-0.65) | 0.62 (0.46-0.78) | **0.80 (0.67-0.91)** | |
| LGG | 513 | 75.6 | 0.75 (0.61-0.86) | | 0.73 (0.57-0.87) | 0.72 (0.56-0.87) | 0.73 (0.59-0.86) | | 0.26 (0.14-0.40) | 0.44 (0.29-0.61) | 0.73 (0.58-0.88) | | 0.37 (0.23-0.51) | 0.65 (0.47-0.83) | **0.77 (0.61-0.90)** | |
| LIHC | 370 | 64.9 | 0.49 (0.35-0.64) | | 0.46 (0.32-0.60) | 0.54 (0.41-0.68) | 0.60 (0.45-0.74) | | 0.54 (0.40-0.67) | 0.60 (0.45-0.75) | 0.53 (0.37-0.68) | | 0.61 (0.49-0.72) | 0.61 (0.47-0.76) | **0.66 (0.52-0.80)** | |
| LUAD | 497 | 63.8 | 0.68 (0.56-0.79) | | 0.69 (0.57-0.80) | 0.66 (0.54-0.77) | 0.75 (0.66-0.85) | | 0.67 (0.57-0.76) | 0.63 (0.51-0.76) | **0.77 (0.68-0.85)** | | 0.70 (0.61-0.78) | 0.72 (0.60-0.82) | 0.74 (0.64-0.84) | |
| LUSC | 489 | 56.6 | 0.58 (0.46-0.69) | | 0.59 (0.47-0.70) | 0.62 (0.51-0.73) | **0.64 (0.53-0.75)** | | 0.54 (0.40-0.66) | 0.52 (0.39-0.65) | 0.63 (0.51-0.74) | | 0.54 (0.40-0.67) | 0.52 (0.38-0.64) | 0.59 (0.49-0.70) | |
| MESO | 85 | 14.1 | 0.65 (0.42-0.85) | | 0.82 (0.63-0.97) | 0.70 (0.47-0.92) | 0.77 (0.56-0.93) | | 0.38 (0.15-0.69) | **0.81 (0.67-0.94)** | 0.72 (0.51-0.92) | | 0.31 (0.11-0.59) | 0.79 (0.63-0.93) | 0.75 (0.51-0.93) | |
| OV | 419 | 37.7 | 0.44 (0.34-0.54) | | 0.53 (0.42-0.65) | 0.56 (0.44-0.67) | 0.56 (0.44-0.68) | | 0.46 (0.35-0.57) | 0.53 (0.41-0.64) | 0.59 (0.47-0.69) | | 0.48 (0.37-0.59) | 0.52 (0.41-0.64) | **0.66 (0.56-0.76)** | |
| PAAD | 178 | 47.8 | 0.50 (0.33-0.69) | | 0.52 (0.36-0.69) | 0.65 (0.45-0.84) | 0.55 (0.38-0.73) | | 0.60 (0.40-0.77) | 0.56 (0.38-0.74) | **0.66 (0.49-0.80)** | | 0.61 (0.42-0.78) | 0.50 (0.31-0.68) | 0.53 (0.37-0.68) | |
| READ | 164 | 84.1 | 0.37 (NA) | | 0.46 (NA) | 0.58 (NA) | 0.51 (NA) | | 0.39 (NA) | 0.34 (NA) | **0.71 (NA)** | | 0.45 (NA) | 0.27 (NA) | 0.66 (NA) | |
| SARC | 259 | 62.2 | 0.55 (0.39-0.74) | | 0.54 (0.37-0.71) | 0.50 (0.32-0.67) | 0.60 (0.43-0.77) | | 0.54 (0.34-0.74) | 0.45 (0.29-0.62) | **0.68 (0.48-0.86)** | | 0.48 (0.31-0.66) | 0.38 (0.23-0.58) | 0.58 (0.41-0.76) | |
| SKCM | 459 | 52.1 | 0.65 (0.55-0.76) | | 0.73 (0.62-0.82) | **0.73 (0.62-0.83)** | 0.65 (0.54-0.76) | | 0.53 (0.41-0.65) | 0.37 (0.26-0.49) | 0.62 (0.49-0.73) | | 0.54 (0.42-0.66) | 0.39 (0.28-0.50) | 0.63 (0.52-0.74) | |
| STAD | 401 | 60.6 | 0.51 (0.38-0.63) | | 0.59 (0.46-0.71) | 0.63 (0.50-0.73) | 0.67 (0.56-0.77) | | 0.61 (0.48-0.74) | 0.54 (0.40-0.69) | 0.61 (0.43-0.80) | | **0.69 (0.57-0.81)** | 0.58 (0.43-0.72) | 0.67 (0.56-0.77) | |
| UCEC | 541 | 83.2 | 0.78 (0.63-0.89) | | 0.73 (0.56-0.86) | 0.72 (0.55-0.86) | **0.81 (0.68-0.90)** | | 0.80 (0.61-0.92) | 0.79 (0.61-0.91) | 0.72 (0.53-0.86) | | 0.79 (0.59-0.91) | 0.73 (0.51-0.88) | 0.81 (0.66-0.91) | |
| UCS | 57 | 38.6 | 0.71 (0.10-1.00) | | 0.75 (0.30-1.00) | 0.41 (0.12-1.00) | 0.35 (0.05-0.71) | | 0.12 (0.00-0.34) | 0.79 (0.36-1.00) | 0.61 (0.15-1.00) | | 0.17 (0.00-0.35) | **0.76 (0.32-1.00)** | 0.34 (0.06-0.67) | |
| UVM | 80 | 58.8 | 0.66 (0.18-1.00) | | 0.54 (0.00-1.00) | 0.72 (0.26-1.00) | 0.71 (0.24-1.00) | | 0.72 (0.32-1.00) | 0.61 (0.20-1.00) | 0.64 (0.19-0.96) | | 0.66 (0.22-1.00) | 0.61 (0.20-1.00) | **1.00 (NA)**^b^ | |
| Mean±SD | - | - | 0.603±0.111 | | 0.634±0.107 | 0.644±0.107 | 0.650±0.125 | | 0.549±0.172 | 0.579±0.155 | 0.658±0.068 | | 0.570±0.155 | 0.592±0.149 | **0.692±0.133** | |

Note: For each cancer type, the best result is shown in bold. Embed: embeddings derived directly from pretrained scFoundation weights; Embed-pan: embeddings from scFoundation fine-tuned by unfreezing the last three encoder layers in pan-cancer training; Clin, clinical data; mRNA, mRNA expression data; EGSP-pan, model integrating Embed-pan, gene expression, and clinical features; EGSP, model integrating Embed, gene expression, and clinical features. ^a^ NA indicates that the confidence interval could not be reliably estimated due to the small number of events and/or high censoring rate in the test cohort. For some cancer types with very small test sets, only point estimates are shown and CIs are omitted. ^b^ The confidence interval was not reported when it degenerated to a single point (e.g., 1.00–1.00) due to very small sample size or limited numbers of comparable pairs, and was therefore considered unreliable.

**Table S6. Performance comparison of pan-cancer training across different numbers of unfrozen layers and input modalities, evaluated using iAUC (95% CI).**

| Unfrozen layers | Embed-pan | |  | Embed-pan+Clin | |
| --- | --- | --- | --- | --- | --- |
|  | Validation set | Test set |  | Validation set | Test set |
| None | 0.775 (0.748–0.802) | 0.744 (0.715–0.772) |  | 0.789 (0.762–0.815) | 0.760 (0.730–0.789) |
| −1 | 0.777 (0.750–0.802) | 0.737 (0.708–0.768) |  | 0.795 (0.769–0.820) | 0.757 (0.728–0.787) |
| −1, −2 | 0.775 (0.745–0.801) | 0.748 (0.719–0.778) |  | 0.788 (0.761–0.815) | 0.752 (0.724–0.783) |
| −1, −2, −3 | 0.780 (0.752–0.806) | 0.741 (0.710–0.771) |  | 0.792 (0.764–0.817) | **0.765 (0.738–0.792)** |
| −1, −2, −3, −4 | 0.782 (0.753–0.807) | 0.740 (0.711–0.771) |  | 0.785 (0.758–0.811) | 0.756 (0.727–0.786) |

Note: Bolded values indicate the highest performance in the test set. Performance in **Table S6** was evaluated using a single pan-cancer model trained on pooled training sets and tested on a pooled pan-cancer test set across all 25 cancer types, and is therefore not directly comparable to the cancer-type–specific mean performance reported in **Table S4**. *Embed-pan* denotes the pan-cancer model using Embed-pan as input, whereas *Embed-pan+Clin* additionally incorporates clinical variables. “Unfrozen layers” refers to the transformer encoder layers in scFoundation that were progressively unfrozen from the top (e.g., *–1* = last layer, *–1, –2* = last two layers, etc.).

**Table S7. Rough comparison of C-indices reported by EGSP and other models.**

| Cancer | Clin+mRNA |  | mRNA | |  | Clin+mRNA | |  | Clin+mRNA | |  | Clin+mRNA+CNV | |  | mRNA+miRNA+CNV  +DNAm+Mut+WSI | |  | mRNA+miRNA  +DNAm+CNV | |  | Clin+mRNA  +miRNA+WSI | |
| --- | --- | --- | --- | --- | --- | --- | --- | --- | --- | --- | --- | --- | --- | --- | --- | --- | --- | --- | --- | --- | --- | --- |
|  | FBSP (ours) |  | Huang et al.[13] | Delta(%) |  | Fan et al.[10] | Delta(%) |  | Cheerla et al.[9] | Delta(%) |  | Fan et al.[10] | Delta(%) |  | CATfusion[12] | Delta(%) |  | DCAP[8] | Delta(%) |  | Cheerla et al.[9] | Delta(%) |
| ACC | **0.833** |  |  |  |  |  |  |  |  |  |  |  |  |  | 0.830 | 0.4 |  |  |  |  |  |  |
| BLCA | 0.681 |  | 0.566 | 20.5 |  | 0.636 | 7.1 |  | 0.58 | 17.5 |  | 0.665 | 2.5 |  | 0.647 | 5.3 |  | 0.646 | 5.5 |  | **0.73** | -6.7 |
| BRCA | 0.746 |  | 0.716 | 4.3 |  | 0.681 | 9.6 |  | 0.56 | 33.3 |  | 0.665 | 12.2 |  | 0.724 | 3.1 |  | 0.662 | 12.8 |  | **0.79** | -5.5 |
| CESC | 0.739 |  | 0.598 | 23.5 |  | 0.703 | 5.2 |  | 0.62 | 19.2 |  | 0.676 | 9.4 |  | 0.742 | -0.4 |  | 0.685 | 7.9 |  | **0.76** | -2.7 |
| COAD | 0.690 |  |  |  |  | 0.596 | 15.8 |  | 0.58 | 19.0 |  | 0.595 | 16.0 |  | 0.722 | -4.4 |  | 0.622 | 11.0 |  | **0.74** | -6.7 |
| ESCA | 0.813 |  |  |  |  |  |  |  |  |  |  |  |  |  | 0.605 | 34.3 |  | 0.594 | 36.8 |  |  |  |
| GBM | 0.627 |  |  |  |  |  |  |  |  |  |  |  |  |  |  |  |  |  |  |  |  |  |
| HNSC | **0.674** |  | 0.592 | 14.0 |  | 0.641 | 5.2 |  | 0.55 | 22.6 |  | 0.642 | 5.0 |  | 0.620 | 8.8 |  | 0.628 | 7.4 |  | 0.67 | 0.7 |
| KIRC | **0.822** |  | 0.685 | 19.9 |  | 0.721 | 13.9 |  | 0.65 | 26.4 |  | 0.708 | 16.0 |  | 0.725 | 13.3 |  |  |  |  | 0.73 | 12.5 |
| KIRP | **0.925** |  | 0.871 | 6.3 |  | 0.817 | 13.2 |  | 0.64 | 44.6 |  | 0.791 | 17.0 |  | 0.804 | 15.1 |  |  |  |  | 0.79 | 17.1 |
| LAML | **0.813** |  |  |  |  | 0.695 | 16.9 |  | 0.61 | 33.2 |  | 0.674 | 20.5 |  |  |  |  |  |  |  | 0.67 | 21.3 |
| LGG | **0.856** |  |  |  |  | 0.817 | 4.8 |  | 0.67 | 27.8 |  | 0.818 | 4.7 |  | 0.849 | 0.8 |  | 0.823 | 4.0 |  | 0.85 | 0.7 |
| LIHC | 0.680 |  | 0.696 | -2.2 |  | 0.639 | 6.5 |  | 0.69 | -1.4 |  | 0.627 | 8.5 |  | 0.577 | 17.9 |  | 0.710 | -4.2 |  | **0.77** | -11.6 |
| LUAD | **0.765** |  | 0.660 | 15.9 |  | 0.636 | 20.2 |  | 0.58 | 31.8 |  | 0.638 | 19.8 |  | 0.667 | 14.6 |  | 0.629 | 21.6 |  | 0.73 | 4.7 |
| LUSC | 0.636 |  | 0.496 | 28.1 |  | 0.598 | 6.3 |  | 0.51 | 24.7 |  | 0.579 | 9.8 |  | **0.651** | -2.3 |  | 0.597 | 6.5 |  | 0.66 | -3.7 |
| MESO | 0.757 |  |  |  |  |  |  |  |  |  |  |  |  |  | 0.609 | 24.3 |  | **0.765** | -1.0 |  |  |  |
| OV | **0.685** |  | 0.570 | 20.1 |  | 0.591 | 15.8 |  | 0.52 | 31.7 |  | 0.584 | 17.2 |  | 0.552 | 24.0 |  |  |  |  | 0.67 | 2.2 |
| PAAD | 0.548 |  | 0.516 | 6.3 |  | 0.637 | -14.0 |  | 0.61 | -10.2 |  | 0.617 | -11.2 |  | 0.569 | -3.7 |  | 0.665 | -17.6 |  | **0.74** | -26.0 |
| READ | 0.589 |  |  |  |  |  |  |  |  |  |  |  |  |  | **0.724** | -18.6 |  |  |  |  |  |  |
| SARC | 0.613 |  |  |  |  |  |  |  |  |  |  |  |  |  | 0.670 | -8.5 |  | **0.719** | -14.7 |  |  |  |
| SKCM | **0.721** |  |  |  |  | 0.644 | 11.9 |  | 0.550 | 31.0 |  | 0.648 | 11.2 |  | 0.692 | 4.1 |  | 0.644 | 11.9 |  | 0.720 | 0.1 |
| STAD | 0.613 |  | 0.507 | 21.0 |  | 0.568 | 8.0 |  | 0.540 | 13.6 |  | 0.581 | 5.6 |  | 0.655 | -6.3 |  | 0.591 | 3.8 |  | **0.780** | -21.3 |
| UCEC | 0.690 |  |  |  |  | 0.704 | -2.0 |  | 0.630 | 9.5 |  | 0.707 | -2.4 |  | 0.729 | -5.4 |  |  |  |  | **0.850** | -18.8 |
| UCS | 0.577 |  |  |  |  |  |  |  |  |  |  |  |  |  | 0.528 | 9.3 |  |  |  |  |  |  |
| UVM | **1.000** |  |  |  |  |  |  |  |  |  |  |  |  |  | 0.748 | 33.7 |  |  |  |  |  |  |
| Mean | 0.724 |  | 0.623 | 14.8 |  | 0.666 | 8.5 |  | 0.594 | 22.0 |  | 0.660 | 9.5 |  | 0.680 | 6.9 |  | 0.665 | 6.1 |  | 0.744 | -2.6 |

Note: Delta refers to the relative performance improvement of EGSP compared to previous methods. For each cancer type, the best result is shown in bold. Clin, clinical data; mRNA, mRNA expression data; miRNA, microRNA expression data; CNV, copy number variation; DNAm, DNA methylation variation; Mut, mutation data; WSI, whole slide images.
